# Longitudinal neuroanatomical and cognitive progression of posterior cortical atrophy

**DOI:** 10.1093/brain/awz136

**Published:** 2019-06-20

**Authors:** Nicholas C Firth, Silvia Primativo, Razvan-Valentin Marinescu, Timothy J Shakespeare, Aida Suarez-Gonzalez, Manja Lehmann, Amelia Carton, Dilek Ocal, Ivanna Pavisic, Ross W Paterson, Catherine F Slattery, Alexander J M Foulkes, Basil H Ridha, Eulogio Gil-Néciga, Neil P Oxtoby, Alexandra L Young, Marc Modat, M Jorge Cardoso, Sebastien Ourselin, Natalie S Ryan, Bruce L Miller, Gil D Rabinovici, Elizabeth K Warrington, Martin N Rossor, Nick C Fox, Jason D Warren, Daniel C Alexander, Jonathan M Schott, Keir X X Yong, Sebastian J Crutch

**Affiliations:** 1 Dementia Research Centre, Institute of Neurology, University College London, 8–11 Queen Square, London, UK; 2 Centre for Medical Image Computing, Department of Computer Science, University College London, Gower Street, London, UK; 3 Department of Human Science, LUMSA University, Via della Traspontina, 21, Rome, Italy; 4 Department of Neurology, University Hospital Virgen del Rocio, Seville, Spain; 5 Memory and Aging Center, University of California San Francisco, San Francisco, California, USA; 6 School of Biomedical Engineering and Imaging Sciences, King's College London, UK

**Keywords:** Alzheimer’s disease, brain atrophy, dementia, structural MRI, memory

## Abstract

Posterior cortical atrophy is a clinico-radiological syndrome characterized by progressive decline in visual processing and atrophy of posterior brain regions. With the majority of cases attributable to Alzheimer’s disease and recent evidence for genetic risk factors specifically related to posterior cortical atrophy, the syndrome can provide important insights into selective vulnerability and phenotypic diversity. The present study describes the first major longitudinal investigation of posterior cortical atrophy disease progression. Three hundred and sixty-one individuals (117 posterior cortical atrophy, 106 typical Alzheimer’s disease, 138 controls) fulfilling consensus criteria for posterior cortical atrophy-pure and typical Alzheimer’s disease were recruited from three centres in the UK, Spain and USA. Participants underwent up to six annual assessments involving MRI scans and neuropsychological testing. We constructed longitudinal trajectories of regional brain volumes within posterior cortical atrophy and typical Alzheimer’s disease using differential equation models. We compared and contrasted the order in which regional brain volumes become abnormal within posterior cortical atrophy and typical Alzheimer’s disease using event-based models. We also examined trajectories of cognitive decline and the order in which different cognitive tests show abnormality using the same models. Temporally aligned trajectories for eight regions of interest revealed distinct (*P* < 0.002) patterns of progression in posterior cortical atrophy and typical Alzheimer’s disease. Patients with posterior cortical atrophy showed early occipital and parietal atrophy, with subsequent higher rates of temporal atrophy and ventricular expansion leading to tissue loss of comparable extent later. Hippocampal, entorhinal and frontal regions underwent a lower rate of change and never approached the extent of posterior cortical involvement. Patients with typical Alzheimer’s disease showed early hippocampal atrophy, with subsequent higher rates of temporal atrophy and ventricular expansion. Cognitive models showed tests sensitive to visuospatial dysfunction declined earlier in posterior cortical atrophy than typical Alzheimer’s disease whilst tests sensitive to working memory impairment declined earlier in typical Alzheimer’s disease than posterior cortical atrophy. These findings indicate that posterior cortical atrophy and typical Alzheimer’s disease have distinct sites of onset and different profiles of spatial and temporal progression. The ordering of disease events both motivates investigation of biological factors underpinning phenotypic heterogeneity, and informs the selection of measures for clinical trials in posterior cortical atrophy.

## Teaser

In the first multicentre, international longitudinal investigation of disease progression in posterior cortical atrophy, Firth *et al.* reveal distinct trajectories of cognitive decline and patterns of tissue loss compared to typical Alzheimer’s disease. Mapping this heterogeneity provides a basis for understanding the factors underlying variability in neurodegenerative disease expression.

## Introduction

Considerable heterogeneity is evident among individuals with Alzheimer’s disease both clinically (e.g. amnesic, visual, dysexecutive/behavioural and aphasic presentations of Alzheimer’s disease) (Galton *et al.*, 2000) and pathologically (e.g. hippocampal sparing and limbic-predominant Alzheimer’s disease subtypes accounting for 25% of cases) (Murray *et al.*, 2011). This heterogeneity provides an important opportunity to explore the factors that promote or inhibit disease progression, both spatially and temporally. One recent example is the discovery of structural variations in amyloid-β fibrils between individuals with rapidly and slowly progressing forms of Alzheimer’s disease (Qiang *et al.*, 2017). In this context, longitudinal quantitative tracking of variations in regional tissue damage in large cohorts of patients with different phenotypic expressions of a disease can reveal the temporal profiles of disease evolution and permit testing of the hypothesis that differentially distributed molecular lesions predict particular patterns of disease progression.

Perhaps the most striking example of heterogeneity in Alzheimer’s disease is seen in posterior cortical atrophy (PCA). PCA is a clinico-radiological syndrome involving a progressive, dramatic and relatively selective decline in higher visual processing and other posterior cortical functions (Benson *et al.*, 1988; Crutch *et al.*, 2012). The condition is most commonly associated with the histopathological features of Alzheimer’s disease, but the distribution of pathology differs from typical Alzheimer’s disease. PCA involves prominent tissue loss in the posterior regions of the brain, with a greater density of neurofibrillary tangles (and to a lesser extent, neuritic plaques) in occipital, posterior parietal and temporo-occipital cortex and fewer pathological changes in more anterior areas such as prefrontal cortex (Hof *et al.*, 1990, 1997; Levine *et al.*, 1993; Ross *et al.*, 1996). A small number of cases of PCA have been attributed to alternative aetiologies including corticobasal degeneration (CBD), Lewy body disease (LBD), and prion disease (Tang-Wai *et al.*, 2003*a*, *b*; Renner *et al.*, 2004). The prevalence and incidence of PCA are not known but age of onset is most commonly in the fifties or sixties (Mendez *et al.*, 2002; Schott *et al.*, 2016). The atypical, predominantly posterior distribution of damage in PCA relative to typical Alzheimer’s disease has been confirmed using a number of structural and functional neuroimaging metrics [e.g. grey matter atrophy (Lehmann *et al.*, 2011), white matter atrophy and altered diffusivity (Migliaccio *et al.*, 2012), and cerebral blood flow (Kas *et al.*, 2011; Lehmann *et al.*, 2016)]. Although certain molecular pathological metrics show little if any difference between mild-to-moderately affected PCA and typical Alzheimer’s disease patients (e.g. widespread amyloid tracer uptake) (Lehmann *et al.*, 2016), comparable CSF-amyloid-β_42_ levels (Ossenkoppele *et al.*, 2015*b*), the pattern of tau tracer binding associates strongly with clinical phenotype (Ossenkoppele *et al.*, 2016). Individuals with PCA and typical Alzheimer’s disease also show distinct patterns of anti-amyloid-β antibodies (Dorothée *et al.*, 2012) and microglial activation patterns (Kreisl *et al.*, 2017), raising the possibility that immunological responses could contribute to shaping Alzheimer’s disease phenotypes. Genetically, apolipoprotein E (*APOE*) ε4 allele status alters PCA risk but with a smaller effect than for typical Alzheimer’s disease (Schott *et al.*, 2006; Snowden *et al.*, 2007) and an exploratory genome-wide association study has identified three candidate genetic risk factors that may be specific to PCA (Schott *et al.*, 2016).

Understanding the temporal changes associated with neurodegeneration requires longitudinal studies, and to date there have been no systematic longitudinal studies of PCA. A small number of longitudinal case reports and case series have described the clinico-radiological progression of the condition (Ross *et al.*, 1996; Goethals and Santens, 2001; Giovagnoli *et al.*, 2009; Kennedy *et al.*, 2012; Chang *et al.*, 2015; Crutch *et al.*, 2017) or the development of specific cognitive deficits [e.g. dyslexia and excessive visual crowding (Yong *et al.*, 2016), dysgraphia (Primativo *et al.*, 2017)]. The only previous longitudinal group study found comparable rates of widespread grey matter loss in patients with PCA and typical Alzheimer’s disease with a mean disease duration of 5 years, but participant numbers were small (17 PCA, 16 typical Alzheimer’s disease) and the time window limited (1-year interval) (Lehmann *et al.*, 2012). A large cross-sectional dataset has been used to make inferences regarding progression, suggesting an early overlap across Alzheimer’s disease phenotypes in temporoparietal and posterior cingulate atrophy and additional involvement of visual association cortices in PCA, a pattern further emphasized in patients at later disease stages (Ossenkoppele *et al.*, 2015*a*).

In this study, we used longitudinal structural imaging and cognitive profiles of individuals with PCA to track long-term trajectories of change via event-based modelling (EBM) and differential equation-based approaches. Continuous trajectories, as well as the ordering of regional volume loss on structural imaging measures were estimated for PCA and typical Alzheimer’s disease relative to a control group. Similar estimations were carried out for trajectories of cognitive decline and ordering of impaired performance on neuropsychological measures. Our hypothesis was that atrophy rates would be highest in phenotype-specific brain regions early in the disease course but subsequently show convergence, with atrophy rates highest across a wider set of contiguous, phenotype non-specific but Alzheimer’s disease-relevant networks as the disease evolves.

## Materials and methods

### Participants

**Figure 1:**
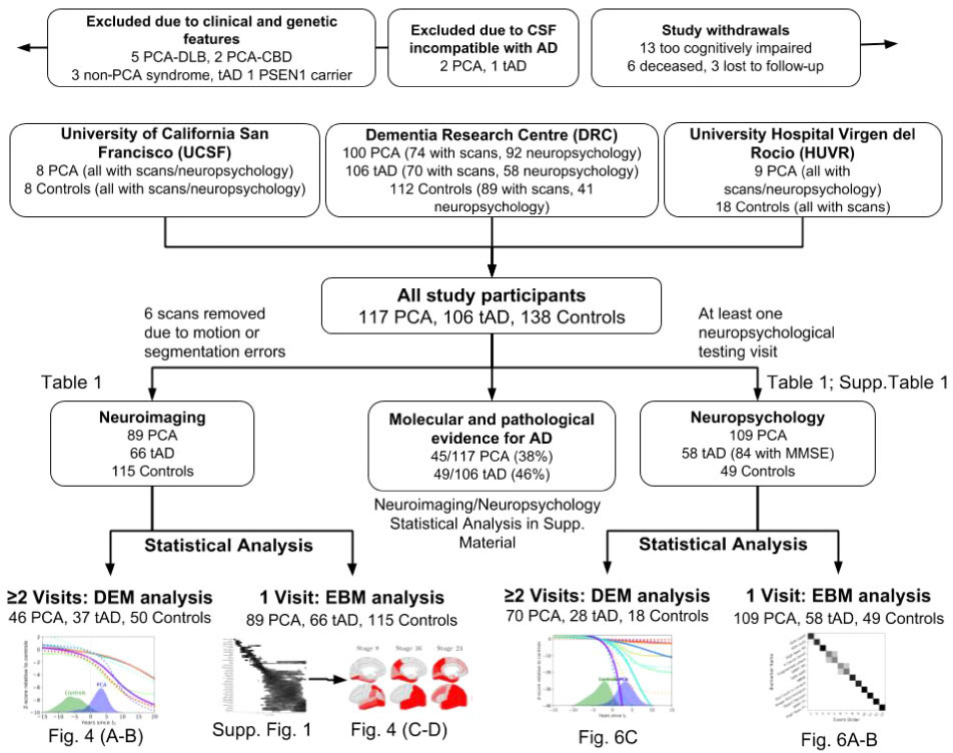
**Study flow chart showing participants included and excluded from analyses.** Study participants were recruited from three different centres from the Dementia Research Centre (DRC), University of California San Francisco (UCSF) and University Hospital Virgen del Rocio (HUVR). Of all the study participants, some underwent neuroimaging (Table 1) and neuropsychological testing (Table 1 and Supplementary Table 1). We performed statistical analysis both longitudinally, using the differential equation model (DEM) and cross-sectionally, using the EBM. Full results are also shown in the Supplementary material on the subset of patients with molecular and pathological evidence of Alzheimer’s disease pathology. AD = Alzheimer’s disease; tAD = typical Alzheimer’s disease; DLB = dementia with Lewy bodies; CBD = corticobasal degeneration; MMSE = Mini-Mental State Examination.

A flow chart of participants included and excluded from the overall study and analyses is shown in Fig. 1. The longitudinal study involved both prospective recruitment from research cohorts, clinics at specialist centres (January 2005 to December 2016) and retrospective case note review (dating back to December 1996). One hundred and seventeen individuals with a clinical diagnosis of PCA were recruited at three specialist centres: 100 patients at the Dementia Research Centre (DRC) at the National Hospital for Neurology and Neurosurgery London (UK), nine patients at the University Hospital Virgen del Rocio (HUVR) Memory Disorders Unit (Spain), and eight patients at the University of California San Francisco (UCSF) Memory and Aging Center (USA). All PCA patients met both Tang-Wai *et al.* (2004) and Mendez *et al.* (2002) criteria based on available information at baseline and expert retrospective clinical review. Prospective participants (*n* = 10) with clinical features of another neurodegenerative syndrome (LBD, CBD, prion disease), were excluded (example features: visual hallucinations, pyramidal signs, reduplicative phenomena, parkinsonism, alien limb syndrome, asymmetric dystonia and myoclonus, ataxia); thus all patients included fulfilled consensus criteria for PCA-pure (Crutch *et al.*, 2017). One hundred and six patients with typical amnestic-predominant Alzheimer’s disease and 138 healthy individuals contributed to patient and control reference samples. All patients with typical Alzheimer’s disease fulfilled clinical criteria for probable Alzheimer’s disease (McKhann *et al.*, 2011); one prospective participant was found to be carrying a presenilin 1 (*PSEN1*) mutation and was excluded. All available molecular or pathological evidence for patients [45/117 (38%) PCA; 49/106 (46%) typical Alzheimer’s disease] supported underlying Alzheimer’s disease pathology [73 had a CSF profile compatible with Alzheimer’s disease (Shaw *et al.*, 2009; Duits *et al.*, 2014) (see Supplementary material for assay-specific cut-offs); six had positive amyloid PET scans; 14 had autopsy-proven Alzheimer’s disease], with the exception of one patient with PCA found to have a CSF profile borderline compatible with Alzheimer’s disease (increased amyloid-β_1–42_ and p-tau, decreased total-tau). In addition to the patients presented above, three patients (two PCA, one typical Alzheimer’s disease) that were initially recruited were excluded from analysis based on their CSF profile not being compatible with underlying Alzheimer’s disease (Duits *et al.*, 2014). Patients with biomarker evidence of Alzheimer’s disease pathology met the McKhann *et al.* (2011) criteria for probable Alzheimer’s disease with high biomarker probability of Alzheimer’s disease aetiology (Dubois *et al.*, 2010). Patients with PCA were followed-up annually until cognitive and/or physical decline prevented further participation (Fig. 1), together with a reference subset of typical Alzheimer’s disease and control participants. Prior ethical approval for the study was provided by the National Research Ethics Service Committee London Queen Square and written informed consent was provided by all participants according to the Declaration of Helsinki.

See Table 1 for demographic details of participants at initial visits and for subsets of participants completing follow-up visits. Of the 361 individuals contributing data to the study, 270 completed at least one neuroimaging assessment and 216 at least one cognitive assessment. From the 270 participants contributing neuroimaging data, a total of 553 scans gathered over a maximum period of 6 years were included in the analysis. From the 216 participants contributing cognitive data, a total of 419 assessments gathered over a maximum period of 6 years were included in the analysis. Participants were well matched for age and patient participants were matched for age at onset at first cognitive assessment. There were more female than male participants in the PCA relative to typical Alzheimer’s disease group (%male: PCA: 39%; typical Alzheimer’s disease: 62%; controls: 50%; *P* < 0.01). Patient groups were matched for Mini-Mental State Examination (MMSE) score at first assessment, with both groups significantly impaired relative to controls (PCA: 20.88 ± 5.17; typical Alzheimer’s disease: 19.38 ± 4.85; controls: 29.02 ± 0.98).

**Table 1:** Demographic details for participants in the study contributing neuroimaging and neuropsychological data

**Total study participants**												
	PCA (*n* = 117)				Typical Alzheimer’s disease (*n* = 106)				Controls (*n* = 138)			
Visits	Number of subjects	Age (SD)	Gender (M:F)	Age at onset (SD)	Number of subjects	Age	Gender (M:F)	Age at onset (SD)	Number of subjects	Age (SD)	Gender (M:F)	Age at onset (SD)
**Neuroimaging**												
** ≥1**	**89**	**63.52 (6.91)**	**55:34**	**58.50 (6.86)**	**66**	**66.39 (8.58)**	**31:35**	**61.71 (8.37)**	**115**	**61.87 (10.43)**	**70:45**	**-**
** **≥2	46	62.11 (6.52)	29:17	58.15 (6.58)	37	66.84 (8.83)	17:20	62.38 (8.52)	50	61.00 (12.01)	31:19	-
** **≥3	31	62.75 (6.50)	19:12	59.16 (6.67)	21	71.00 (6.97)	10:11	65.86 (7.45)	28	65.75 (5.96)	16:12	-
** **≥4	15	61.46 (4.44)	11:4	58.73 (4.61)	14	70.89 (6.33)	8:6	66.93 (6.67)	17	66.82 (4.88)	10:7	-
** **≥5	9	61.73 (4.06)	7:2	58.56 (4.72)	4	72.08 (4.81)	3:1	68.75 (4.87)	8	66.11 (4.83)	3:5	-
** **≥6	2	62.35 (1.65)	1:1	58.50 (1.50)	1	79.90 (0.00)	1:0	76.00 (0.00)	115	61.87 (10.43)	70:45	-
**Neuropsychology**												
** ≥1**	**109**	**64.49 (7.54)**	**41:68**	**59.99 (8.08)**	**58**	**65.68 (7.57)**	**36:22**	**54.74 (4.93)**	**49**	**63.12 (5.90)**	**12:37**	**-**
** **≥2	70	63.64 (7.32)	23:47	59.35 (7.72)	28	64.58 (7.08)	13:15	54.08 (4.78)	18	60.00 (5.87)	0:18	-
** **≥3	45	62.73 (7.26)	16:29	58.70 (7.53)	5	66.08 (2.78)	2:3	56.00 (0.00)	0	-	-	-
** **≥4	20	63.19 (7.00)	7:13	59.84 (6.46)	0	-	-	-	0	-	-	-
** **≥5	7	59.44 (4.84)	2:5	56.25 (4.62)	0	-	-	-	0	-	-	-
** **≥6	2	57.22 (3.49)	1:1	55.32 (1.32)	0	-	-	-	0	-	-	-

Details are presented at initial visit (in bold) and for subsets of participants who completed follow-up assessments (up to a maximum of six). Number of individuals at each assessment, mean (and standard deviation, SD) age, gender and mean age at onset (and standard deviation) are shown per number of visits. F = female; M = male.

### Procedures

T_1_-weighted volumetric magnetic resonance scans were acquired on five different scanners [two 3 T Trio (DRC and UCSF), 1.5 T Intera (HUVR), and two 1.5 Signa units (DRC)] using spoiled gradient recalled or gradient echo (MPRAGE) sequences. The scans consisted of full brain coverage coronal or sagittal slices running between 124 and 208 contiguous slices of 1.5 or 1.0 mm. Full details of imaging parameters are shown in the Supplementary material, and site and scanner distribution in earlier and later PCA are shown in the Supplementary material.

To estimate regional brain volumes, we used the Geodesic Information Flows (GIF) algorithm (Cardoso *et al.*, 2012) to perform tissue segmentation and parcellation. The Neuromorphometrics atlas was used for segmentation, which produced 144 different brain regions of interest across the left and right hemisphere. Segmentation failed for six scans belonging to five subjects (three controls, one PCA and one typical Alzheimer’s disease) due to motion artefacts; these scans were subsequently removed and not included in the statistics in Table 1. Brain volumes were corrected for total intracranial volume (TIV), age and gender, scanner type (1.5 T, 3 T) and site (DRC, UCSF, HUVR) using a general linear model, where gender, scanner type and site were encoded using one-hot encoding. A total of 52 brain regions of interest were removed (18 were not part of the cerebral cortex, six had segmentation errors, 28 were grouped into larger regions of interest). Left and right brain regions were averaged into one region, providing a total of 46 regions of interest, which were further averaged into eight regions of interest corresponding to whole brain, hippocampal, occipital, frontal, entorhinal, temporal (excluding hippocampal), parietal and ventricle volumes.

Participants in the prospective study phase completed an annual battery of neuropsychological tests. This general neuropsychological battery (see Lehmann *et al.*, 2011 for detailed references) includes standard tests of general cognitive function (Supplementary Table 1). Individual test administration was discontinued at subsequent visits for participants scoring at floor, in which case a floor score was assigned for this test at next assessment, with scores at all following assessments classified as missing data.

### Statistical analysis

**Figure 2:**
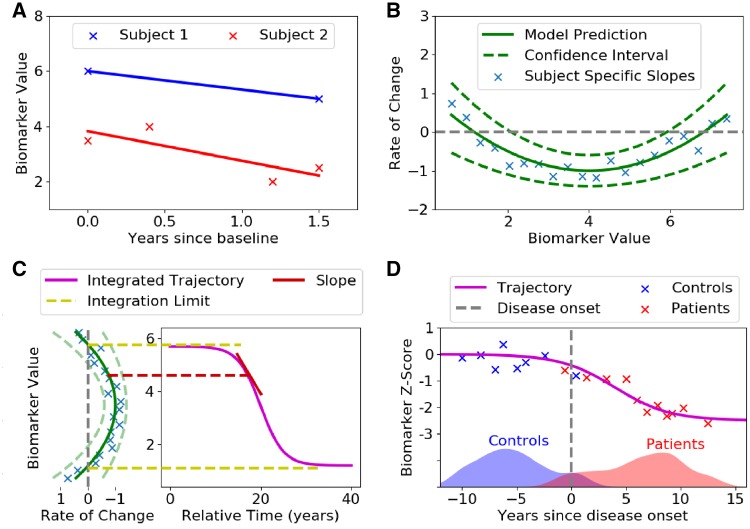
**Diagram of the differential equation model.** (**A**) Measuring biomarker rate of change from line of best fit. The biomarker measurements for each subject were plotted against time since baseline, and a line was fit for each subject independently. The slope of these lines was then used as a measure of the biomarker rate of change. (**B**) Rate of change model. The slopes of each fitted line were plotted against the average biomarker value of each subject (blue crosses). A non-parametric model (Gaussian process regression, green line) was then fitted on measurements, which gave a model prediction and also a 95% confidence interval. (**C**) Trajectory reconstruction. A line integral was performed on the rate of change model from **B**. The integration limits were defined as the biomarker values where the corresponding change is zero or at the limits of the data. Starting from the upper integration limit, the trajectory was reconstructed from the rate of change prediction, which represents the slope corresponding to that biomarker value. Before integration, an arbitrary starting time point, t_0_ = 0, was defined, thus all time is relative to t_0_. (**D**) Anchoring process. In the absence of a reliable estimate of time since disease onset, the origin t_0_ was set as the point that best separates controls from patients, which have been staged along the time axis using their biomarker data. Moreover, to make trajectories comparable across biomarkers we convert the biomarker values to Z-scores with respect to controls, which results in a scaling along the *y*-axis. The process (**A**–**D**) was repeated for each biomarker independently. After fitting each biomarker, the subjects can be staged along the disease timeline, as in (**D**), using the trajectories from all biomarkers.

The differential equation model (DEM) (Villemagne *et al.*, 2013) was used to estimate long-term neuroanatomical and cognitive biomarker trajectories from multiple short-term longitudinal data. For each region of interest or cognitive test the DEM calculates the rate of change for each individual across each of the visits (Fig. 2A). The rate of change of a biomarker was assumed to be a function of the mean biomarker value for each participant. In order to make minimal assumptions on the subsequent trajectory shape, a non-parametric Gaussian process regression model was then fit to all of the participants’ values to give an average rate of change for all biomarker values (Fig. 2B). This function was then integrated, giving an average biomarker value as a function of time (Fig. 2C). With no reliable means of measuring age at onset, biomarker trajectories were aligned by defining a reference time t_0_ (t = 0; Fig. 2D) as the threshold that best separated controls from patients. See the Supplementary material for full DEM method. All participants with more than one assessment were included in these analyses.

To estimate disease progression from limited datasets and to validate the DEM trajectories, we used the EBM (Fonteijn *et al.*, 2012). The EBM derives a probabilistic ordering in which biomarkers show detectable abnormality. The most likely sequence provides a staging system expressing the most likely position of a subject along the most likely sequence of events. In this work, two formulations of the EBM were used corresponding to MRI brain volume data and cognitive test data. For the EBMs using MRI data, the previously published formulation of the model was used. For cognitive data models of normal and abnormal biomarker distributions were represented using kernel density estimation instead of simple parametric distributions used previously in the EBM. See Supplementary material for complete formulations of both EBMs. All available data at baseline visit were included in these analyses.

For DEM results, we tested for differences in estimated biomarker values between time points (−10, 0, and 10 years from t_0_) both within- and between-group. Within-group differences in estimated biomarker values were assessed using two-tailed paired *t*-tests for all pairs of biomarkers (e.g. within PCA, values of biomarker *i* versus biomarker *j* for all *i,j*) at −10, 0, and 10 years since t_0_. Between-group (PCA versus typical Alzheimer’s disease) differences in estimated biomarker values were assessed using two-tailed two sample *t*-tests for all biomarkers at each time point. For EBM results, we tested for statistically significant differences in the estimated abnormality sequences, both within and between groups. Non-parametric tests were used because of non-Gaussianity of the data (data are ordinal representing ranks) and samples were thinned (1 every 100) as adjacent samples in Markov Chain Monte Carlo (MCMC) sampling are correlated. Within-group differences were assessed using Wilcoxon signed-rank one-tailed tests for all pairs of biomarkers *(i,j*), which assesses whether biomarker *i* becomes abnormal before biomarker *j.* Significant differences in the relative position of a biomarker within the EBM abnormality sequence between groups (PCA versus typical Alzheimer’s disease) were assessed using two-tailed Mann-Whitney U-tests. We applied Bonferroni-corrected thresholds for all tests performed on EBM and DEM results.

### Data availability

The data that support the findings of this study are available from the corresponding author, upon reasonable request. All the algorithms used in the present paper are reported in the Supplementary material.

## Results

**Figure 3:**
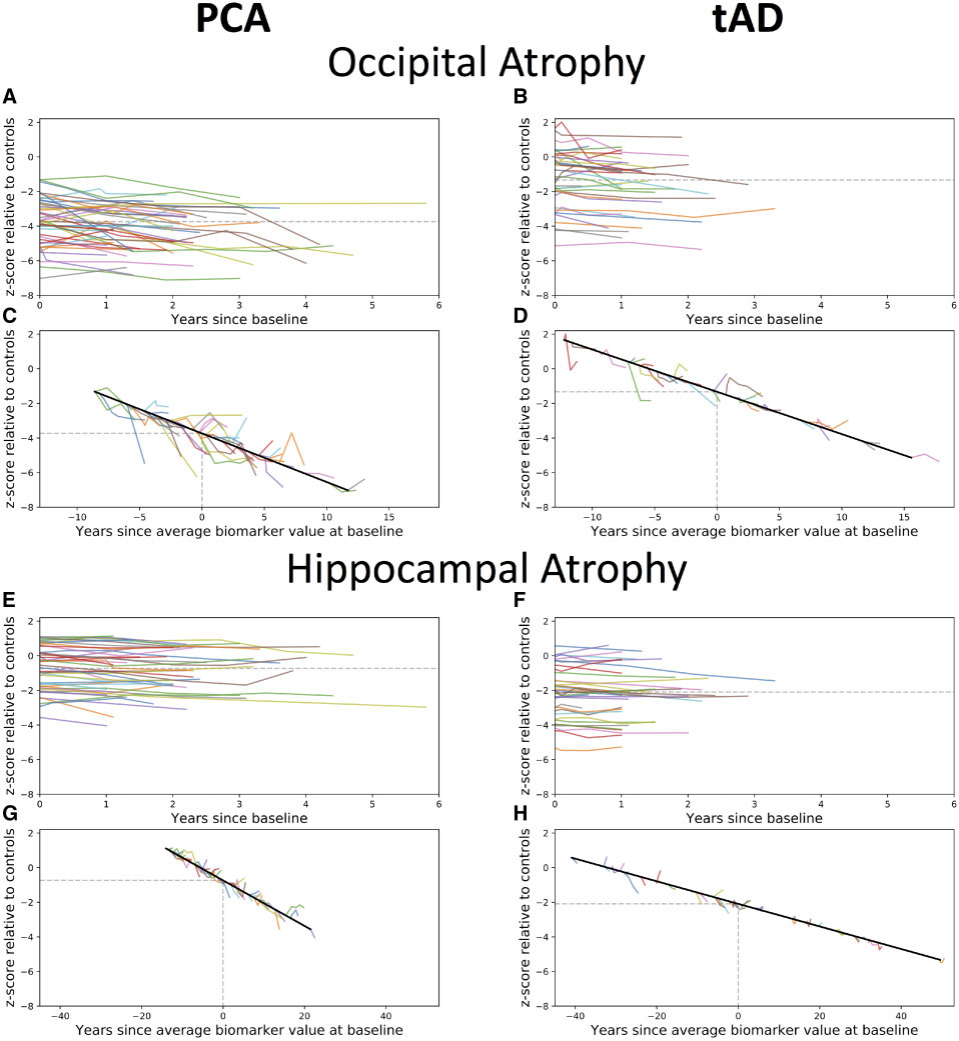
**Observed longitudinal occipital (A–D) and hippocampal (E–H) atrophy, relative to controls, for PCA (*left*) and typical Alzheimer’s disease patients (*right*).** (**A**–**B** and **E**–**F**) Spaghetti plots anchored at baseline visit. (**C**–**D** and **G**–**H**) Hairy line plots for observed longitudinal data anchored to the group trajectory using the baseline value. tAD = typical Alzheimer’s disease.

To give the reader a sense of the raw neuroimaging data, we show in Fig. 3 longitudinal data for two exemplar metrics—occipital and hippocampal atrophy. Compared to patients with typical Alzheimer’s disease, PCA patients showed more occipital (PCA: mean volume z-score = −3.74; typical Alzheimer’s disease: −1.33, *P* < 1 × 10^−9^) and less hippocampal atrophy (PCA: z- score = −0.74; typical Alzheimer’s disease: z = −2.09, *P* < 1 × 10^−4^) at baseline.

**Figure 4:**
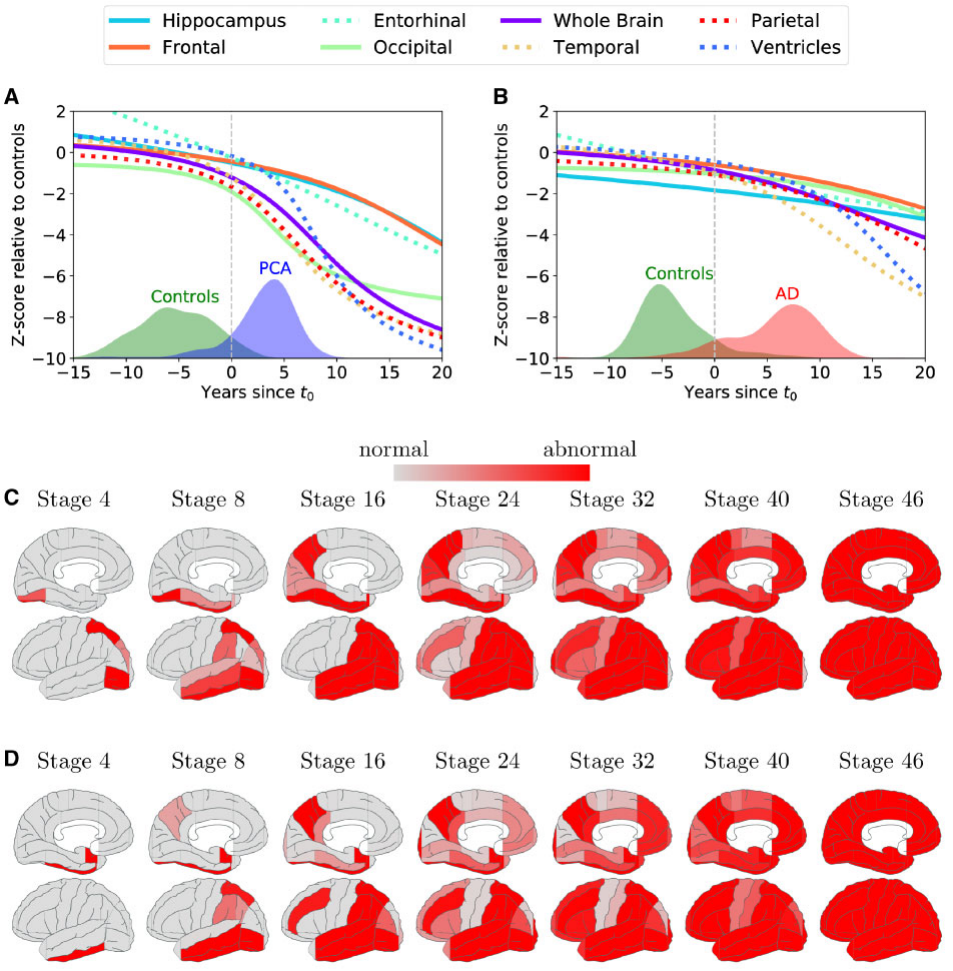
**Region of interest trajectories and ordering of atrophy.** (**A** and **B**) Trajectories of different region of interest volumes from the DEM for (**A**) PCA progression and (**B**) typical Alzheimer’s disease (AD) progression. The *x*-axis shows the number of years since t_0_, and the *y*-axis shows the Z-score of the region of interest volume relative to controls. The trajectories of the ventricles have been flipped to aid comparison. Overlaid are histograms of subject stages based on the estimated trajectories. (**C** and **D**) Ordering of atrophy in (**C**) PCA patients and (**D**) typical Alzheimer’s disease patients according to the EBM. White regions are within the volume range of healthy controls, while red regions are abnormal by the corresponding stage, with shading indicating the probability of abnormality. By stage k, a number of k biomarkers shaded in red became abnormal. For positional variance diagrams used to generate brain figures and full details on methodology, see the Supplementary material.

**Figure 5:**
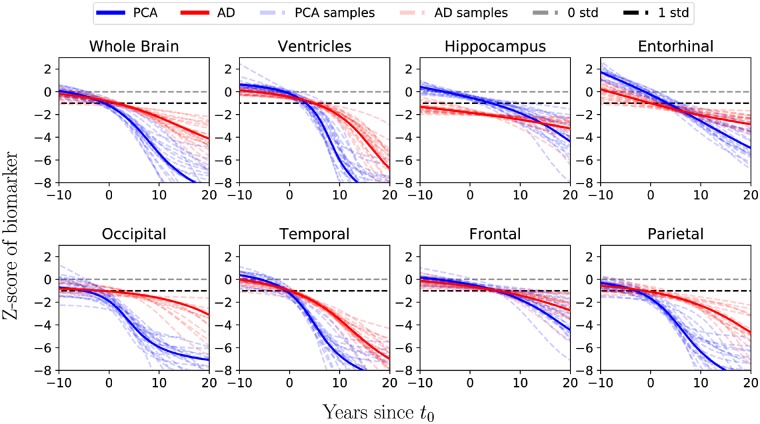
**Mean trajectories for region of interest volumes for PCA and typical Alzheimer’s disease aligned on the same temporal scale with samples from the posterior distribution showing the confidence of the mean trajectory.** The *x*-axis shows the number of years since t_0_, and the *y*-axis shows the z-score of the region of interest volume relative to controls. The trajectories for the ventricles have been flipped to aid visual comparison. AD = Alzheimer’s disease.

Temporally-aligned average DEM trajectories for eight regions of interest were calculated for participants with PCA (Fig. 4A) and typical Alzheimer’s disease (Fig. 4B). Confidence estimates using bootstraps for each average trajectory were also calculated (Fig. 5). Amongst patients with PCA, occipital and parietal atrophy was most evident before t_0_, and by t_0_ we also observe considerable atrophy in the temporal lobe. Across the 10 years following t_0_, we observe a marked increase in the rate of occipital, parietal and temporal atrophy and ventricular expansion. By contrast, hippocampal, entorhinal and frontal atrophy never match the extent of tissue loss in posterior and temporal regions. More than 10 years from t_0_, atrophy rates in occipital, parietal and temporal lobes seem to slow down, but limited data in this time window prevent the drawing of any clear conclusions.

By contrast, before t_0_ patients with typical Alzheimer’s disease showed most extensive tissue loss in the hippocampus, with subsequent rates of change highest for temporal atrophy and ventricular expansion. It should be noted that within 12 years from t_0_, model estimates of parietal and ventricular abnormality amongst patients with typical Alzheimer’s disease are equivalent to or exceed the relative extent of hippocampal abnormality. Comparing PCA and typical Alzheimer’s disease trajectories directly at t_0_ (Fig. 5), the estimated region of interest volumes were lower in posterior regions (parietal: *P* < 1 × 10^−6^; occipital: *P* < 2 × 10^−3^) in PCA relative to typical Alzheimer’s patients overall. On the other hand, participants with PCA had higher hippocampal and entorhinal estimated volumes (both *P* < 1 × 10^−9^) and lower ventricular volume (*P* < 2 × 10^−3^) compared to patients with typical Alzheimer’s disease. Comparison of estimated frontal or temporal region of interest volumes at t_0_ did not find evidence of differences between patient groups following Bonferroni correction (both *P* > 2 × 10^−3^). For a summary of the statistical tests including comparisons 10 years before and following t_0,_ see Supplementary Table 9.

Differences between PCA and typical Alzheimer’s disease progression are also recapitulated with the EBM. Figure 4C and D shows, for both PCA and typical Alzheimer’s disease, snapshots of brain atrophy at model stages 4, 8, 16, 24, 32, 40, and 46 (of 46) generated from the positional variance diagrams (Supplementary Fig. 1). Patients with PCA showed early atrophy in occipital (inferior, anterior, superior, lateral) and superior parietal areas, whilst patients with typical Alzheimer’s disease showed early atrophy in the amygdala, hippocampus and entorhinal cortex, followed by temporal areas. The ordering is largely preserved under bootstrapping and supported by statistical testing (Supplementary Figs 2 and 3). Comparing PCA and typical Alzheimer’s disease abnormality sequences provided evidence of differences in ordering of disease events between patient groups (Supplementary Table 9). Most occipital regions were estimated to become abnormal significantly earlier in PCA compared to typical Alzheimer’s disease, with the exception of the occipital pole, which was estimated to become abnormal earlier in typical Alzheimer’s disease. Temporal, frontal, hippocampus and entorhinal regions were estimated to become abnormal significantly earlier in typical Alzheimer’s disease compared to PCA. There was mixed evidence of parietal regions differing in estimated ordering of abnormality between patient groups. For example, superior parietal and precuneus regions were estimated to become abnormal significantly earlier in PCA and typical Alzheimer’s disease, respectively, although test effect sizes were small. Full statistical testing can be found in Supplementary Fig. 4.

A summary of all available baseline cognitive data is shown in Supplementary Table 1. A number of MMSE scores were available in typical Alzheimer’s disease participants who did not undergo the full neuropsychological assessment (*n* = 31), but are included for disease staging purposes. Both patient groups showed evidence of diminished performance on all of the available cognitive measures relative to controls. The two patient groups had comparable scores on MMSE, visual episodic memory and working memory. Nonetheless, consistent with standard phenotypic descriptions, notable cognitive patterns could be observed as differences in performance between the two groups. Overall, PCA patients exhibited significantly poorer performance in numeracy, basic vision, space and object perception relative to their typical Alzheimer’s disease counterparts. Overall, patients with typical Alzheimer’s disease had a lower performance in verbal episodic memory compared to PCA patients.

**Figure 6:**
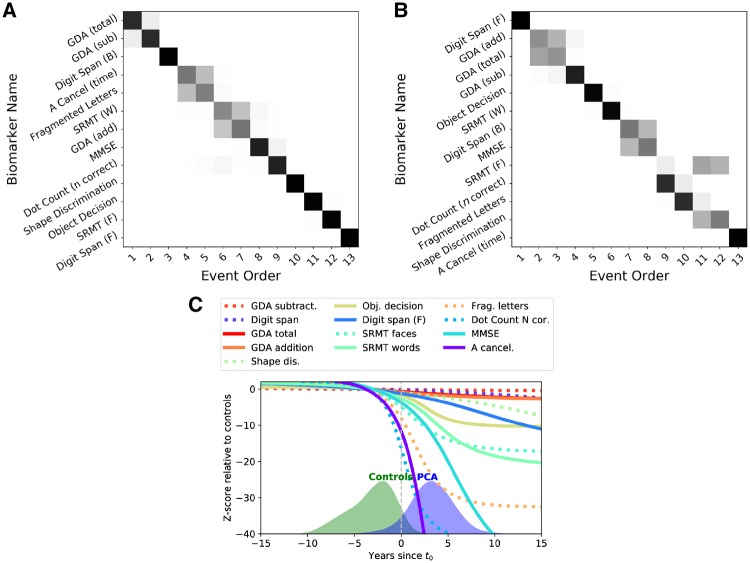
**(**A** and **B**) Positional variance diagrams for (**A**) PCA and (**B**) typical Alzheimer’s disease showing estimated order of impairment on 14 cognitive metrics (*y*-axis) across different stages (positions on *x*-axis).** Each entry (*x,y*) represents the probability of a particular cognitive metric becoming abnormal at a given position in the sequence (darker shades = higher probability). (**C**) Trajectories of different cognitive tests from the differential equation model for PCA progression. The *x*-axis shows the number of years since t_0_, and the *y*-axis shows the z-score on each cognitive test relative to controls. Overlaid are also the histograms of the subjects, as they have been staged by the model.

**Figure 7:**
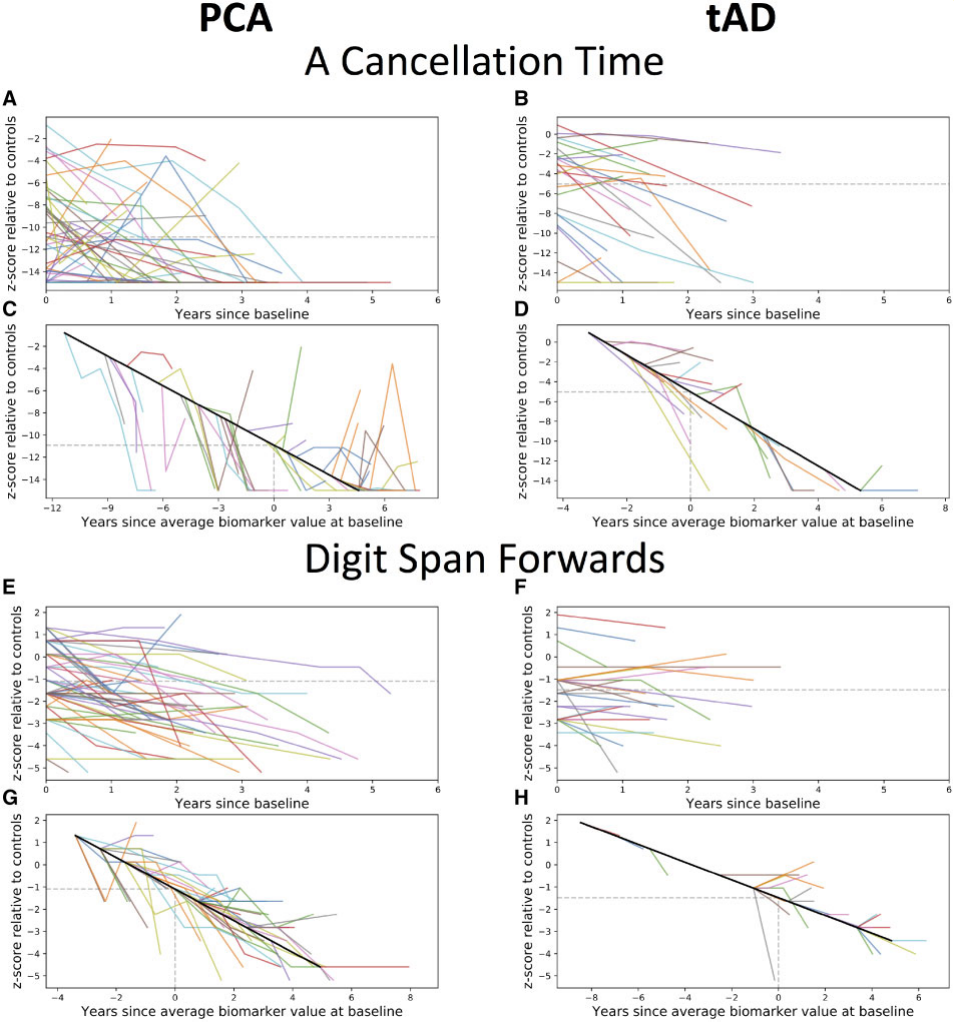
**Observed longitudinal data from example cognitive tasks.** Observed longitudinal A cancellation (**A**–**D**) and digit span forwards (**E**–**H**) scores, relative to controls, for PCA (*left*) and typical Alzheimer’s disease patients (*right*). (**A**–**B** and **E**–**F**) Spaghetti plots anchored at baseline visit. (**C**–**D** and **G**–**H**) Hairy line plots for observed longitudinal data anchored to the group trajectory using the baseline value. tAD = typical Alzheimer’s disease.

To evaluate the cognitive changes due to disease, an EBM maximum likelihood sequence of biomarker abnormality was estimated for both PCA and typical Alzheimer’s disease using 13 cognitive test baseline measures (Fig. 6A and B). Uncertainty in these orderings was estimated using a bootstrapping procedure (Supplementary Fig. 5) (Young *et al.*, 2014). Patients with PCA showed early impairment on perceptual, spatial, numeracy (GDA subtraction) and working memory tasks (digit span backwards). Patients with typical Alzheimer’s disease showed early deficits in working memory, verbal episodic memory and numeracy (addition and subtraction), with impairment on visual tasks a consistently later feature (4/5 visual/visuomotor tasks occupying the latest positions in the sequence). The most notable differences between the PCA and typical Alzheimer’s disease maximum likelihood sequences were the occurrence of the spatially demanding A cancellation time measure (PCA: event 4/13; typical Alzheimer’s disease: event 13/13; *P* < 7 × 10^−4^) and the working memory measure of digit span forwards (PCA: event 13/13; typical Alzheimer’s disease: event 1/13; *P* < 7 × 10^−4^). These measures were selected as exemplar cognitive tasks; corresponding individual observed longitudinal data outline the variability of longitudinal performance on cognitive measures (Fig. 7). Compared to patients with typical Alzheimer’s disease, PCA patients showed significantly poorer A cancellation (PCA: z-score = −10.91; typical Alzheimer’s disease: −5.03; *P* < 1 × 10^−5^) and comparable digit span forward performance (PCA: z-score = −1.10; typical Alzheimer’s disease: z = −1.48; *P* > 0.05). Differences between PCA and typical Alzheimer’s disease sequences of cognitive changes were also statistically significant (Supplementary Figs 7 and 8).

Average trajectories for 13 cognitive measures were calculated for participants with PCA (Fig. 6C). Trajectories for typical Alzheimer’s disease were not calculated due to insufficient follow-up visits from these participants. Confidence estimates for each average trajectory were also calculated (Supplementary Fig. 11). Fragmented letters, dot counting and A cancellation metrics showed greatest impairment at t_0_. These measures also showed the greatest subsequent rates of change, likely reflecting both progression of cognitive impairment but also the psychometric properties of the tests. A second cluster of tasks showing comparable rates of decline included MMSE, shape discrimination, object decision and visual and verbal episodic memory. Of the remaining tasks, working memory measures showed later impairment and lower rates of decline.

## Discussion

This first major longitudinal investigation of disease progression in PCA revealed distinct patterns of tissue loss as compared to typical Alzheimer’s disease. Following the early neuroanatomical signature of occipital and parietal atrophy characterized in previous cross-sectional studies, individuals with PCA underwent further atrophy in those regions as well as temporal lobe atrophy and ventricular expansion. These changes were commensurate in relative extent with occipital and parietal damage within an estimated 10 years of t_0_. By contrast, entorhinal, hippocampal and frontal regions underwent a lower rate of change which, whilst notably abnormal, did not result in the relative magnitude of loss seen even initially in the key posterior regions. Although in PCA there was markedly greater parietal than hippocampal atrophy throughout the disease course, the reverse was not true for typical Alzheimer’s disease; in these individuals the relative extent of parietal atrophy matched that of the hippocampal regions within 11 years of t_0_.

Overall, findings are consistent with a space of continuous phenotypic variability across the full spectrum of Alzheimer’s disease, as is increasingly being recognized both clinically (Dickerson *et al.*, 2011) and pathologically (Murray *et al.*, 2011; Whitwell *et al.*, 2012).

The findings of this study support the idea that the spatial and temporal profiles of PCA and typical Alzheimer’s disease phenotypes may be the consequence of insults originating at different sites within a common disease-relevant network of brain regions (Warren *et al.*, 2013). Findings suggest selective intracortical vulnerability, with early abnormalities estimated for some (e.g. occipital: inferior/superior/middle/fusiform) but not all regions (occipital pole) for the PCA group overall, consistent with relative preservation of visual acuity in PCA (Lehmann *et al.*, 2011). Regions considered key sites of pathological activity in Alzheimer’s disease, such as the entorhinal cortex and hippocampus, exhibit neuroanatomical distinctions between PCA and typical Alzheimer’s disease; such distinctions are evident not only early in the disease, but throughout the disease course. Accordingly, serious consideration must be given to whether trajectories are dictated by a combination of site of origin, relevant connectivity and time, or alternatively by protective factors which reduce the vulnerability of certain networks. For example, between 0 and 10 years from t_0_, PCA subjects revealed more pronounced extent and rate of atrophy overall and within phenotype-specific regions of interest compared to typical Alzheimer’s disease, which suggest that factors other than site of origin or brain connectivity might also be involved. Similarly, the longitudinal clinical data and model-based estimation of change over a near 30-year window mean such distinctions cannot be dismissed as minor variations in early disease expression, as could have been argued or hypothesized previously from solely cross-sectional data.

A key strength of the study is the ability to compare and contrast atrophy and cognitive change between individuals with PCA and typical Alzheimer’s disease. The DEM can infer long-term progression from short-term longitudinal data, which is critical in the case of a sporadic syndrome like PCA, which is relatively rare and cannot practically be identified and followed in asymptomatic individuals. The DEM, unlike the EBM, includes time in the model to permit the estimation of rates of change, although the approach can only integrate the rate of change to a trajectory of data where the modelled average rate of change does not alternate between increasing and decreasing. Another unique advantage of this study is the EBM’s capacity to estimate longitudinal patterns from sufficient, representative cross-sectional data.

The results on neuropsychological data highlight differences and similarities between patients with PCA and typical Alzheimer’s disease in terms of both early cognitive difficulties and trajectories. At the early stages both patient groups demonstrated widespread cognitive decline compared to controls and the two groups did not differ in terms of MMSE, working memory and visual episodic memory, which might be comparably impaired but with different underlying cognitive mechanisms. The differences between the two groups included lower performance in numeracy, visuoperceptual and visuospatial processing in PCA relative to typical Alzheimer’s disease. In contrast, lower performance in verbal episodic memory was observed in typical Alzheimer’s disease compared to PCA patients. The different pattern of results observed for visual and verbal episodic memory suggest that the former involve an important component of visual processing and might represent an unreliable measure of episodic memory in PCA. Longitudinally, the data highlighted different trajectories of decline for different cognitive domains in the two groups of patients. Among the most striking differences, performance on measures sensitive to visuospatial processing (such as the A cancellation test) was estimated to decline early in PCA and relatively late in typical Alzheimer’s disease. Conversely, performance on measures of working memory (such as the forward digit span) were estimated to decline early in typical Alzheimer’s disease and late in PCA. An apparently unexpected result may relate to the backward digit span being estimated as becoming abnormal relatively early in PCA. The backward digit span is indeed a measure of working memory. Nonetheless, the recent literature on cognitive psychology and neuroimaging suggests that the backward digit span is a complex cognitive test, which requires the activation of multiple cognitive systems and brain circuits. In particular, the prominent contribution of visuospatial imagery and the high activation of the dorsolateral prefrontal cortex have been shown (Rapport *et al.*, 1994; Reynolds *et al.*, 1997; Hoshi *et al.*, 2000; Hilbert *et al.*, 2014). The literature thus seems to suggest that caution must be placed when interpreting the cognitive processes underlying this test, since cognitive functions other than working memory, some of which are already known to be impaired in PCA, might cause a decline in performance.

While the current study represents the largest longitudinal investigation of PCA to date comprising analyses of structural imaging and comprehensive neuropsychological measures, several limitations should be noted. Owing to the relative rarity of PCA, data were acquired over an extended time period; for the majority of this period (from 1996 onwards), evidence for underlying Alzheimer’s disease pathology was only routinely available at autopsy. Correspondingly, amyloid PET imaging and CSF were only available in a proportion of patients, with non-Alzheimer’s disease aetiologies (DLB, CBD) ruled out based on available clinical information. Nevertheless, repeating analyses on the subset of patients with evidence of underlying Alzheimer’s disease pathology generated findings that were consistent with overall results (Supplementary Figs 9–17). Another limitation of our study is that imaging data were acquired on different scanners of different strengths, although these covariates were regressed out after the estimation of regional brain volumes. Moreover, another limitation is the comparatively small amount of data collected from sites outside of the UCL Dementia Research Centre, thus limiting our understanding of PCA outside of this centre. Imbalanced numbers from different sites is a limitation in many multicentre studies, in particular in studies in which cognitive tests are administered in different languages. Controlling for differences across centres becomes increasingly challenging with smaller numbers in specific centres as unintended variance may be removed by regressing out differences between centres. We hope that improvements in the diagnosis of PCA will promote more studies spanning different countries and socioeconomic areas, and more large-scale analyses to incorporate these data. While numbers of time points varied between participants, the EBM and DEM approaches outlined above enabled the estimation of ordering of disease events and non-linear trajectories based on cross-sectional and participant-specific short-term longitudinal data. These approaches have characteristic assumptions that are important to consider when interpreting the current findings. First of all, the DEM estimates every trajectory independently, so after DEM fitting we need to put multiple trajectories on a common axis, which also requires normalization and a time anchor. Here we used the threshold that best separates controls from patients to anchor the time (t = 0). The DEM trajectories are also susceptible to floor and ceiling effects in both brain volumes and cognitive measures, but the probabilistic nature of the DEM goes some way to ameliorating this. With regards to the EBM, it assumes trajectories are step-functions, where biomarkers switch from a normal to an abnormal value. Finally, both the EBM and DEM estimate a population-average disease progression, which is not necessarily indicative of how each individual will progress.

Future studies will need to focus on disentangling the genetic, pathological, molecular and neurodevelopmental factors responsible for the distinction in long-term disease pathways and progression reported in this paper. For example, pilot genome-wide association study findings in PCA implicate genes associated with intercellular communication, signalling pathways related to retinal degeneration and the development of the visual system, but these results must be replicated in larger samples and the impacts of heterogeneity and young age at onset be further disambiguated. Building on the insights of the landmark investigations 20 years ago (Hof *et al.*, 1997, 1990), neuropathological studies must also examine the relative impact of disease upon specific neuronal populations and different inflammatory processes. It is hoped that better understanding the causes of phenotypic heterogeneity in Alzheimer’s disease may prove a catalyst for novel therapeutic strategies.

## Supplementary Material

awz136_Supplementary_Data

## Abbreviations

DEM: differential equation model
EBM: event-based model
PCA: posterior cortical atrophy
