# Supplementary material for: Longitudinal neuroanatomical and cognitive progression of posterior cortical atrophy
Source: Brain. 2019 Jun 20;142(7):2082–95. doi: 10.1093/brain/awz136 (PMC6598737; doi:10.1093/brain/awz136)
Supplement: awz136_Supplementary_Data [file awz136_supplementary_data.pdf]

Supplementary Table 1. Baseline neuropsychology data. Number of participants (*n*) and mean and standard deviation (SD) score are given for each group. P values according to the Mann-Whitney U test, between PCA and typical Alzheimer's disease, PCA and controls, typical Alzheimer's disease and control groups are reported.

	Max score	**PCA**		**tAD**		**Control**		**PCA vs tAD**	**PCA vs Control**	**tAD vs Control**
		n	Mean (SD)	*n*	Mean (SD)	*n*	Mean (SD)			
MMSE	30	104	20.88 (5.17)	84	19.38 (4.85)	48	29.02 (0.98)	NS	p<0.001	p<0.001
Episodic memory										
Visual (Short Recognition Memory Test for faces)	25	63	18.75 (4.12)	37	18.76 (4.69)	23	24.61 (0.94)	NS	p<0.001	p<0.001
Verbal (Short Recognition Memory Test for words)	25	98	19.91 (3.79)	38	16.21 (3.38)	23	24.52 (0.85)	p<.001	p<0.001	p<0.001
Working memory										
Digit Span (forwards)	12	77	6.0 (2.96)	56	6.23 (2.17)	23	8.78 (1.73)	NS	p<0.001	p<0.001
Digit Span (backwards)	12	67	3.39 (1.86)	57	3.98 (2.37)	23	7.96 (1.49)	NS	p<0.001	p<0.001
Numeracy										
GDA (addition)	12	58	1.31 (2.23)	51	2.47 (2.7)	23	7.17 (3.14)	p<0.001	p<0.001	p<0.001
GDA (subtraction)	12	58	0.91 (1.95)	51	2.06 (2.92)	23	7.0 (3.85)	p<0.05	p<0.001	p<0.001
Basic vision										
Shape detection (VOSP)	20	103	15.92 (3.66)	57	18.26 (1.77)	0	N/A	p<0.001	-	-
Shape discrimination	20	85	14.78 (3.38)	55	17.73 (3.05)	22	19.41 (0.8)	p<0.001	p<0.001	p<0.05

Crowding	10	78	8.29 (3.16)	55	10.00 (0.00)	0	N/A	p<0.001	-	-
Space perception										
Number location (VOSP)	10	103	2.82 (3.01)	55	5.35 (4.02)	0	N/A	p<0.001	-	-
Dot counting (VOSP)	10	88	4.92 (3.36)	54	8.26 (2.64)	23	9.91 (0.29)	p<0.001	p<0.001	p<0.001
A cancellation (time)	90	99	79.23 (31.78)	55	44.19 (21.4)	31	19.87 (4.66)	p<0.001	p<0.001	p<0.001
A cancellation (n missed)	21	101	4.68 (4.85)	55	0.93 (1.56)	31	0.16 (0.37)	p<0.001	p<0.001	p<0.005
Object perception										
Object decision (VOSP)	20	107	10.37 (4.58)	56	16.34 (2.38)	31	18.06 (1.65)	p<0.001	p<0.001	p<0.001
Fragmented letters (VOSP)	20	79	5.15 (5.49)	55	14.36 (6.07)	22	19.5 (0.67)	p<0.001	p<0.001	p<0.001
Unusual Views	20	68	4.29 (4.64)	51	10.92 (5.16)	0	N/A	p<0.001	-	-
Usual Views	20	68	12.54 (6.24)	51	18.2 (2.37)	0	N/A	p<0.001	-	-

N/A= not available

NS = the difference is not statistically significant

MMSE = Mini Mental State Examination (Folstein et al, 1975)

GDA = Graded Difficulty Arithmetic test (Jackson & Warrington, 1986)VOSP = Visual and object space perception test (Warrington and James, 1991)

Supplementary Methods

Imaging parameters

T1-weighted volumetric MR scans were acquired on five different scanners at three sites:

Site 1 - DRC:

1.5T scanners: Scans from two 1.5T scanners were included in this study, both of which used an inversion recovery (IR)-prepared SPGR with a 256x256 image matrix and a field of view of 24cm to provide 124 contiguous 1.5mm coronal slices. Acquisition parameter for scanner #1: TE=6.3ms, TR=14.2ms, TI=650ms, flip angle=15°; parameters for scanner #2: TE=5.2ms, TR=12ms, TI=650ms, flip angle=13°.

3T scanner: Siemens Trio TIM 3T scanner which used an MPRAGE sequence with a 256x256 acquisition matrix and 28.2-cm field of view to provide 208 contiguous 1.1mm slices in the sagittal plane; acquisition parameters: TE=2.9ms, inversion interval TR=2200ms, TI=900ms.

Site 2 - UCSF:
Siemens Trio TIM 3T scanner which used an MPRAGE sequence; acquisition parameters: TE=2.98ms, TR=2300ms, TI=900ms, flip angle=15°, slice thickness 1.5mm.

Site 3 - HUVR:
1.5T Philips Intera scanner which used a TFE T1 3D sequence with 288x288 acquisition matrix; acquisition parameters: TE=4ms, TR=8.69ms, flip angle=8°, slice thickness 1mm.

Cerebrospinal fluid (CSF) assays/analysis

CSF samples were acquired at two sites, using two assays and assay-specific cut-offs for underlying AD pathology:

Site 1 - DRC:
Samples were analyzed for amyloid-β1-42 and total-tau using INNOTEST enzyme-linked immunosorbent assays. Applied cut-offs for this site were as follows: amyloid- β1-42 ≤ 627 pg/ml and/or total-tau/amyloid- β ratio >0.52 (Duits et al., 2014).

Site 2 - UCSF:
Samples were analyzed for amyloid-β1-42, total-tau and p-tau using INNO-BIA AlzBio3 Fujirebio multiplex Luminex assay. The applied cut-off for this site was a total-tau/ amyloid- β ratio >0.39 (Shaw et al., 2009).

Event-Based Model

The event-based model (EBM) was introduced by [1] and describes the disease progression as a sequence of discrete events, where each event corresponds to a biomarker value switching from normal to abnormal. The aim of the model is to find the most likely ordering in which events occur given a dataset of biomarker measurements.

The EBM has the following mathematical formulation. We define a series of events $E_1, E_2, \ldots, E_N$ where each event E_k corresponds to a biomarker k switching from normal values (representative of healthy controls) to abnormal values (representative of late stage patients). N is the number of biomarkers used. For modelling disease progression, we also define an ordering $S = [s(1), \ldots, s(N)]$, which is a permutation of the biomarkers 1, . . . , N creating the event ordering $E_{s(1)}, E_{s(2)}, \ldots, E_{s(N)}$. The set of events is specified a-priori and corresponds to the set of biomarkers used in the dataset. The model uses a dataset X which contains a set of X_j measurements for each subject j. Each set X_j =

$\{x_{1j}, x_{2j}, \ldots, x_{Nj}\}$ where x_{ij} represents the value of biomarker *i* in subject *j* and is informative of event E_i in subject *j*. If a subject j is at stage $k \in [0, .., N]$ in the disease progression, events $E_{s(1)}, \ldots, E_{s(k)}$ have occurred while events $E_{s(k+1)}, \ldots, E_{s(N)}$ have not occurred. We can therefore define the likelihood of the data from subject *j* at stage *k* given ordering *S* as:

$$p(X_j|S,k) = \prod_{i=1}^{k} p\left(x_{s(i),j}|E_{s(i)}\right) \prod_{i=k+1}^{N} p\left(x_{s(i),j}|\neg E_{s(i)}\right)$$

Where $p(x_{ij}\,|E_i)$ represents the likelihood of measuring x_{ij} given event E_i occurred by stage *k*, while $p(x_{ij}\,|not\ E_i)$ represents the likelihood of measuring x_{ij} given event E_i has not occurred by stage *k*. For estimating a model for $p(x|not\ E_i)$ we fit a Gaussian distribution on data from healthy controls, while for estimating $p(x|E_i)$ we fit a uniform distribution on all observed biomarker measurements. In order to get the full likelihood of the dataset *X* given sequence *S*, we marginalize over stage *k* of every subject, which is unknown, and assume independence between measurements from other biomarkers and subjects:

$$p(X|S) = \prod_{j=1}^{J}\left[\sum_{k=0}^{N} p(k)\left(\prod_{i=1}^{k} p\left(x_{s(i),j}|E_{s(i)}\right) \prod_{i=k+1}^{N} p\left(x_{s(i),j}|\neg E_{s(i)}\right)\right)\right]$$

where *J* is the total number of subjects.

The full likelihood is maximized using a greedy search, by continuously applying small perturbations to the sequence *S* and selecting the perturbed sequences that maximise the likelihood. A posterior over the possible sequences *S* given *X* is estimated using Markov-chain Monte Carlo sampling.

Non-parametric EBM for cognitive data

Kernel Density Estimation (KDE) is a non-parametric method of probability density estimation, that is useful for data smoothing. The KDE estimation, $\hat{f}(x)$, of a function, $f(x)$, with an independent and identically distributed sample, $x_1, x_2, \ldots, x_n$, drawn from a distribution with an unknown density, is given by

$$\hat{f}(x) = \frac{1}{nh}\sum_{i} K\left(\frac{x - x_i}{h}\right)$$

where K is non-negative function, which integrates to one and has mean zero, and h is a positive smoothing factor called a bandwidth. With an appropriate choice of K, KDE naturally extends to multivariate density estimation.

In this work we use the scikit-learn (Pedregosa et al. 2011) implementation of KDE, using default parameters, including Gaussian kernel, for all values except the bandwidth, which was estimated using Scotts normal reference rule (Scott 1979).

To allow for a more accurate fitting of mixture models with unknown underlying distributions we have used a non-parametric mixture modelling. Let $x_i \in \mathbb{R}^m$ be a set of observations, and $S_1^{(1)}, S_{21}^{(1)}, \ldots, S_n^{(1)}$ be known subsets of the data. The bandwidth h of these data is then estimated by applying Scotts rule to all the observations. Mixture weights $m_1^{(1)}, m_2^{(1)}, \ldots, m_n^{(1)}$ are initiated as $1/k$ for all subsets. Similarly to the *k*-means algorithm, the Kernel Density Mixture Model (KDEMM) algorithm then iterates over an assignment and update steps to optimise parameters.

Update Step

For each subset of the data, $S_j^{(t)}$, a KDE mixture j component, $\hat{f}_{S_j^{(t)}}$ is fit using

$$\hat{f}_{S_j^{(t)}}(x_i) = \frac{m_j^{(t)}}{h} \sum_{x_i \in S_j} K\left(\frac{x_i - x_j}{h}\right)$$

Assignment Step

Each observation is then assigned to a new subset, $S_j^{(t+1)}$, to which it has the maximum likelihood of j belonging, i.e. $\hat{f}_{S_j^{(t)}}(x_i)$. Mixture weights are then updated to be the proportion of observations in each subset i.e. $m_j^{(t+1)} = \left|S_j^{(t+1)}\right| / \sum_{i=1}^{k}\left|S_j^{(t+1)}.\right|$

The update and assignment steps are then iterated until subset assignment is no longer updated, i.e. $S_j^{(t)} = S_j^{(t+1)} \,\forall\, j$. In this work an additional constraint, $0.1 < m_j < 0.9 \,\forall\, j$, is placed on the mixture weights to ensure that subsets do not vanish, similarly to the Gaussian Mixture Model.

Differential Equation Model

The differential equation model (DEM) [2, 3, 4] constructs biomarker trajectories
from the change in biomarker values between different visits. In many datasets such as ADNI,
the biomarker scores *s* are observed for each subject over a few visits. By determining how
these scores change (*Δs*) over time (*t*) during a specified time interval *Δt*, the temporal rate of progression (*Δs/Δt*) can be modelled as a function of the mean biomarker value *f(s) = Δs/Δt*. We model *f(s)* using Gaussian Process (GP) regression using a squared exponential kernel, because it's stationary, infinitely smooth and works well in practice.

After estimating f(s) using GP regression, we then perform a line integral along f(s) to recover s(t). More explicitly, if we take the limit as Δ → 0 we get that:

$$lim_{\Delta t \to 0} \frac{\Delta s}{\Delta t} = \frac{\delta s}{\delta t} = f(s)$$

We solve this numerically using the Euler method. We set an initial (t_0, s_0), a small increment step δt and we find the next pair (t_1, s_1) as follows:

$$t_1 = t_0 + \delta t$$
$$s_1 = s_0 + f(s_0)\delta t$$

This is the repeated until the full trajectory defined by $(t_0, s_0), (t_1, s_1), \ldots, (t_n, s_n)$ is reconstructed. The process is repeated independently for the other biomarkers. Note that t_0 referred to in this section is not related to the group separation point defined later.

Statistical Testing

Statistical Methods

For DEM results, we tested for differences in estimated biomarker values at timepoints -10, 0 and 10 years from group separation point t_0, both within- and between-group. Within-group differences in

estimated biomarker values were assessed using two-tailed paired t-tests for all pairs of biomarkers between pairs of timepoints (-10 vs 0, 0 vs 10, 10 vs -10). Between-group (PCA vs tAD) differences in estimated biomarker values were assessed using two-tailed two sample t-tests for all biomarkers at each timepoint. We applied Bonferroni-corrected thresholds for all tests performed on EBM and DEM results.

For EBM results, we tested how confident we are that a biomarker *i* becomes abnormal before another biomarker *j* both within- and between-group. We used non-parametric tests due to non-gaussianity of the data (data is ordinal representing ranks). Within-group differences were assessed using Wilcoxon signed-rank one-tailed tests for all pairs of biomarkers *(i,j)*. Between-group (PCA vs tAD) differences were assessed using two-tailed Mann-Whitney U tests.

Statistical analysis has been done to evaluate differences between diseases and within the same disease. The following sections provide details for statistical testing for results with (1) the event-based model (EBM) and (2) differential equation model (DEM).

Results of Statistical testing

Event-Based Model

In the EBM analysis, we tested for differences in positions of biomarkers in EBM sequence in two ways: (a) within the same clinical group (e.g. within tAD) and (b) across different clinical groups (e.g. PCA vs tAD).

Statistical testing within same clinical group

Supplementary Figure 3 show the results of statistical testing for significant differences in PCA, tAD on neuroimaging data. Similar results are shown for cognitive tests in Supplementary Figure 7. Each black square represents a pair of biomarkers *(i,j)* where the null hypothesis was rejected at 95% confidence after Bonferroni correction. We observe that most of the squares above the diagonal are black – we are confident that early biomarkers indeed become abnormal before the later ones. Only some pairs of biomarkers that are close to each other in the sequence did not pass the test, suggesting uncertainty is too large to draw any meaningful conclusions about their ordering.

Statistical testing across PCA vs tAD

For every biomarker, we tested for significant differences in the position of that biomarker in the sequence samples from PCA vs tAD. We applied a non-parametric two-tailed Mann-Whitney U test, again due to the fact that data is ordinal. We computed p-values and used bonferroni-corrected significance levels.

Supplementary Figure 4 shows the results of performing statistical testing for comparing PCA vs tAD using neuroimaging data. Regions that become abnormal significantly earlier in PCA compared to tAD include occipital areas, ventricles and the superior parietal lobe, whereas temporal areas become abnormal earlier in tAD. Supplementary Figure 8 shows similar results using cognitive tests, with significant differences observed across all biomarkers. The statistically significant differences between PCA and tAD suggest it is very likely that the two underlying populations are different.

Differential Equation Model

We tested for significant differences in estimated biomarker values at three timepoints: -10, 0 and 10 years from group separation point t_0. We calculated these differences (a) across pairs of biomarkers *(i, j)* within the same clinical group (e.g. within PCA) and (b) for every biomarker across different clinical groups (i.e. PCA vs tAD). In all situations, we applied two-tailed t-tests at 95% significance levels, which were Bonferroni corrected for all comparisons made.

Supplementary Tables 2-4 show the p-values for differences in PCA trajectories at -10, 0 and 10 years from t_0, while Supplementary Tables 5-7 show p-values for differences in tAD trajectories at -10, 0 and 10 years respectively. Significant differences that passed Bonferroni correction are shown with (*). At PCA timepoint t_0, we observe significant differences in parietal vs non-posterior regions, or between occipital vs non-occipital regions. On the other hand, at tAD timepoint t=0 we have significant differences between hippocampus vs other regions, but some of these differences become non-significant after 10 years from disease t_0. These results suggest that within both PCA and tAD, not all regions progress the same, and there are significant differences between phenotype-specific regions versus other regions at different timepoints along the disease.

When comparing biomarker trajectories between PCA and tAD (Supplementary Table 8), we find that 10 years before t_0 there are significant differences in ventricles, hippocampus and entorhinal cortex, and by t_0 all regions apart from temporal and frontal show significant differences. Finally, after 10 years from t_0, we notice that entorhinal cortex doesn't show significant differences anymore, but instead the temporal lobe shows significant differences. Results suggest that there are statistically significant differences between PCA and tAD not just at t_0, but across the entire disease timeline.

Neuroimaging Supplementary Results

Ordering of abnormality – Event Based Model

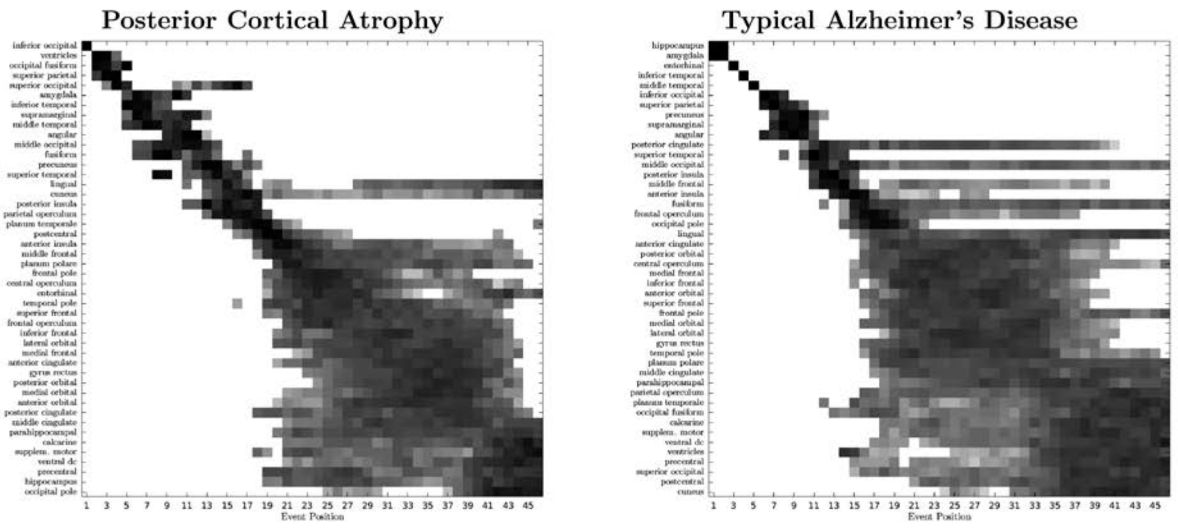

Supplementary Figure 1. Positional variance diagram of the most likely atrophy sequence, as estimated by the event-based model, for (left) PCA progression and (right) tAD progression. The ROIs on the Y-axis are ordered according to the timing of abnormality, from early abnormalities (top labels) to late abnormalities (bottom labels). The X-axis shows the position of a biomarker in the

abnormality sequence. Each pixel at position *(i,j)* shows the probability of biomarker j becoming abnormal at position I, with darker squares showing higher confidence and whiter squares showing lower confidence. The biomarker orderings are sampled from the EBM posterior distribution.

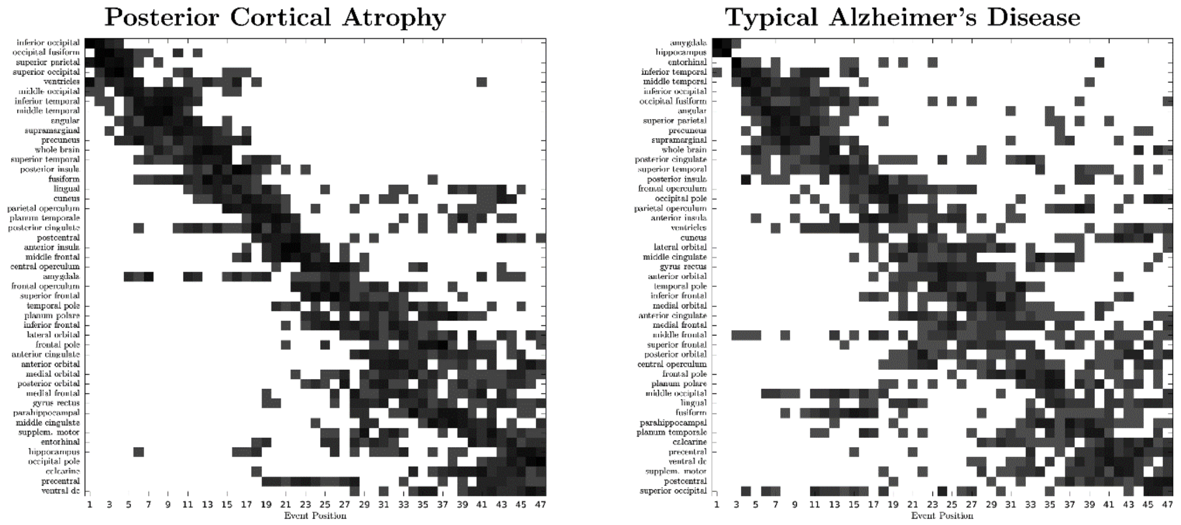

Supplementary Figure 2: Bootstrap samples of the atrophy sequence as estimated by the event-based model, for the PCA and typical AD cohorts. The maximum likelihood sequences were estimated using the EBM from 100 bootstrap datasets, where subjects were sampled with replacement, keeping the same proportion of subjects per diagnostic category.

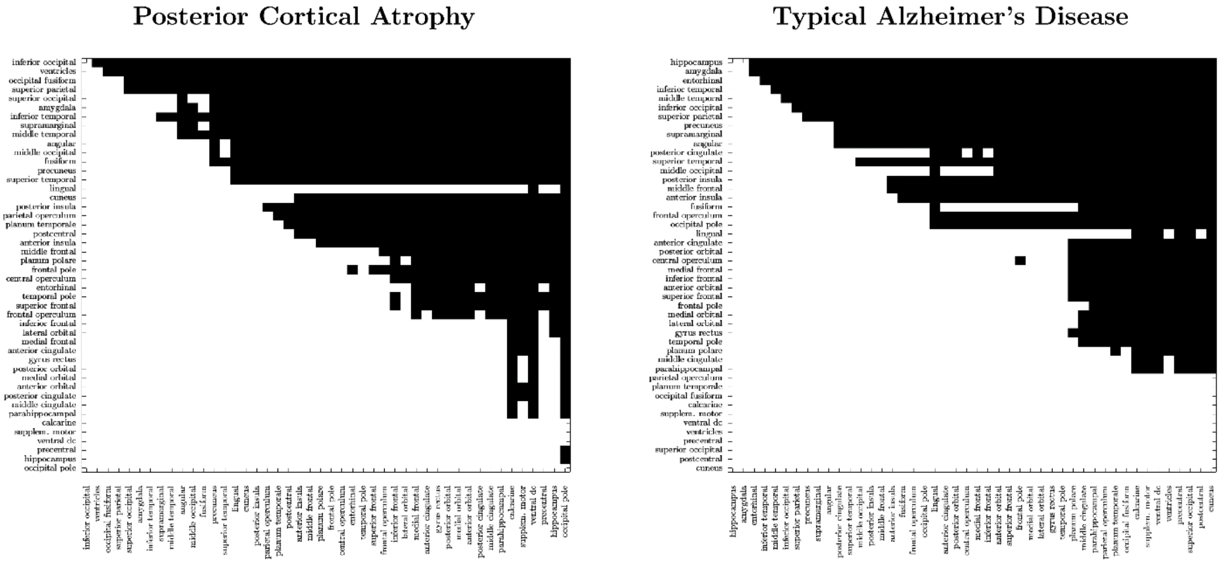

Supplementary Figure 3: Hypothesis testing of ordering of events within PCA (left) and typical AD (right) for the event-based model (EBM). We sampled 10,000 sequences from the EBM posterior using MCMC sampling and thinned them (i.e. only kept every 1/100) to remove correlated samples. We applied the non-parametric paired Wilcoxon signed rank test for every pair of biomarkers *(x, y)*. The null hypothesis is defined as H0: event A (Y-axis) becomes abnormal at the same time as event B (X-axis), while the alternative hypothesis H1: event A (Y-axis) become abnormal before event B (X-axis). The black squares show the pair of biomarkers where the null hypothesis was rejected at

alpha=0.05/(N*(N-1)/2), thus surviving Bonferroni correction. Given that most squares above the diagonal are black, we are confident that, the brain regions are affected in this particular order for PCA and tAD, given the EBM assumptions.

Comparison between diagnostic groups

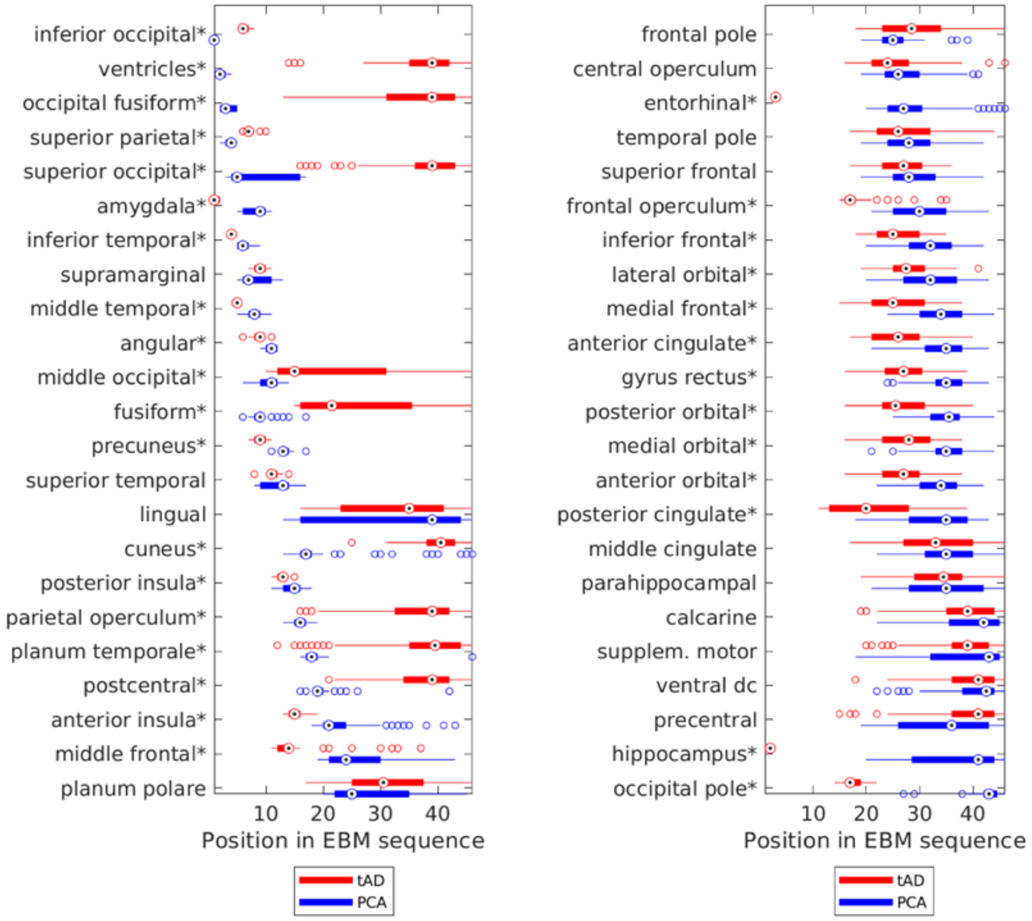

Supplementary Figure 4: Testing for differences in positions of each biomarker in the EBM abnormality sequences, for both PCA and typical AD. (*) Statistically significant differences in position of a biomarker in the EBM sequences for PCA and tAD at 95% confidence, Bonferroni corrected for multiple comparisons (significance level = 2e-04). A non-parametric Mann-Whitney U test has been applied because of non-gaussianity of the data, which represents discrete ranks in a sequence. All biomarkers apart from lingual show significant differences – it is likely that there are differences in atrophy progression between PCA and tAD.

Continuous Trajectory Estimation – Differential Equation Model

Region	Whole Brain	Ventricles	Hippocampus	Entorhinal	Occipital	Temporal	Frontal	Parietal
Whole Brain	-	-	-	-	-	-	-	-
Ventricles	1.74e-04*	-	-	-	-	-	-	-
Hippocampus	1.20e-02	4.95e-02	-	-	-	-	-	-
Entorhinal	1.61e-12*	1.27e-06*	5.29e-10*	-	-	-	-	-
Occipital	7.93e-03	4.16e-06*	1.20e-04*	9.44e-12*	-	-	-	-
Temporal	2.66e-01	1.17e-02	3.12e-01	5.90e-10*	1.81e-03	-	-	-
Frontal	9.58e-01	1.57e-04*	1.07e-02	1.52e-12*	8.68e-03	2.49e-01	-	-
Parietal	3.45e-04*	1.31e-08*	2.52e-07*	3.17e-15*	8.84e-01	7.91e-05*	4.08e-04*	-

Supplementary table 2 Statistical testing for significant differences in volumes of different brain regions of PCA subjects at -10 years before disease t_0. Shown here are p-values from two-tailed t-tests. (*) Statistically significant differences at significance level = 1.78e-3, Bonferroni corrected for all 28 comparisons.

Region	Whole Brain	Ventricles	Hippocampus	Entorhinal	Occipital	Temporal	Frontal	Parietal
Whole Brain	-	-	-	-	-	-	-	-
Ventricles	1.52e-16*	-	-	-	-	-	-	-
Hippocampus	6.03e-13*	8.95e-06*	-	-	-	-	-	-
Entorhinal	4.78e-14*	5.66e-01	9.60e-04*	-	-	-	-	-
Occipital	1.32e-06*	3.17e-17*	1.45e-14*	5.25e-16*	-	-	-	-
Temporal	3.57e-01	1.75e-16*	5.22e-13*	2.90e-14*	1.66e-05*	-	-	-
Frontal	7.31e-12*	1.67e-04*	7.72e-01	4.38e-03	1.62e-14*	3.50e-12*	-	-
Parietal	1.53e-07*	1.41e-21*	3.30e-19*	2.68e-19*	2.20e-01	8.33e-06*	3.39e-18*	-

Supplementary table 3. Statistical testing for significant differences in volumes of different brain regions of PCA subjects at disease t_0. See Supp. Table 1 for information on statistical testing.

Region	Whole Brain	Ventricles	Hippocampus	Entorhinal	Occipital	Temporal	Frontal	Parietal
Whole Brain	-	-	-	-	-	-	-	-
Ventricles	5.97e-01	-	-	-	-	-	-	-
Hippocampus	7.63e-13*	4.14e-13*	-	-	-	-	-	-
Entorhinal	5.88e-11*	2.34e-11*	1.23e-03*	-	-	-	-	-
Occipital	4.04e-02	1.44e-01	3.00e-17*	1.06e-15*	-	-	-	-
Temporal	2.83e-03	1.22e-02	1.51e-15*	2.66e-14*	1.54e-01	-	-	-
Frontal	8.90e-15*	5.73e-15*	6.77e-02	7.35e-07*	2.19e-19*	2.99e-17*	-	-
Parietal	7.38e-02	2.07e-01	1.25e-14*	4.00e-13*	9.44e-01	1.73e-01	1.91e-16*	-

Supplementary table 4. Statistical testing for significant differences in volumes of different brain regions of PCA subjects at 10 years after t_0. See Supp. Table 1 for information on statistical testing.

Region	Whole Brain	Ventricles	Hippocampus	Entorhinal	Occipital	Temporal	Frontal	Parietal
Whole Brain	-	-	-	-	-	-	-	-
Ventricles	9.26e-03	-	-	-	-	-	-	-
Hippocampus	2.04e-10*	2.88e-14*	-	-	-	-	-	-
Entorhinal	2.21e-02	3.40e-01	6.82e-09*	-	-	-	-	-
Occipital	3.38e-03	2.01e-06*	3.84e-04*	9.98e-04*	-	-	-	-
Temporal	3.93e-01	1.04e-01	4.72e-11*	7.64e-02	7.51e-04*	-	-	-
Frontal	8.30e-01	1.04e-02	4.79e-09*	2.41e-02	1.15e-02	3.26e-01	-	-
Parietal	4.94e-03	2.13e-06*	3.75e-05*	4.63e-04*	7.35e-01	8.64e-04*	1.57e-02	-

Supplementary table 5. Statistical testing for significant differences in volumes of different brain regions of tAD subjects at -10 years before t_0. See Supp. Table 1 for information on statistical testing.

Region	Whole Brain	Ventricles	Hippocampus	Entorhinal	Occipital	Temporal	Frontal	Parietal
Whole Brain	-	-	-	-	-	-	-	-
Ventricles	3.50e-11*	-	-	-	-	-	-	-
Hippocampus	4.12e-19*	3.61e-25*	-	-	-	-	-	-
Entorhinal	7.83e-02	2.64e-10*	2.93e-13*	-	-	-	-	-
Occipital	7.65e-02	2.51e-08*	7.95e-10*	7.94e-01	-	-	-	-
Temporal	6.29e-03	4.63e-13*	1.84e-14*	5.12e-01	8.07e-01	-	-	-
Frontal	2.01e-04*	2.65e-04*	4.16e-20*	2.84e-05*	1.98e-04*	3.31e-07*	-	-
Parietal	3.56e-03	6.00e-11*	1.94e-10*	2.45e-01	4.77e-01	4.81e-01	2.12e-06*	-

Supplementary table 6. Statistical testing for significant differences in volumes of different brain regions of tAD subjects at t_0. See Supp. Table 1 for information on statistical testing.

Region	Whole Brain	Ventricles	Hippocampus	Entorhinal	Occipital	Temporal	Frontal	Parietal
Whole Brain	-	-	-	-	-	-	-	-
Ventricles	2.92e-02	-	-	-	-	-	-	-
Hippocampus	2.83e-01	1.67e-03*	-	-	-	-	-	-
Entorhinal	8.13e-03	6.50e-01	2.63e-04*	-	-	-	-	-
Occipital	8.40e-02	9.92e-01	1.13e-02	7.28e-01	-	-	-	-
Temporal	2.76e-12*	1.46e-14*	5.41e-11*	6.35e-15*	2.54e-10*	-	-	-
Frontal	1.24e-09*	1.91e-06*	3.87e-11*	4.81e-06*	1.27e-04*	2.43e-19*	-	-
Parietal	7.92e-01	7.53e-02	1.98e-01	2.51e-02	1.57e-01	5.36e-11*	3.13e-08*	-

Supplementary table 7. Statistical testing for significant differences in volumes of different brain regions of tAD subjects at 10 years after t_0. See Supp. Table 1 for information on statistical testing.

Region	T1 = -10 years	T2 = 0 years	T3 = 10 years
Whole Brain	2.52e-02	4.45e-05*	4.19e-12*
Ventricles	3.74e-05*	3.06e-05*	2.18e-13*
Hippocampus	6.15e-15*	1.13e-23*	5.83e-04*
Entorhinal	5.28e-08*	7.71e-11*	2.68e-03
Occipital	5.72e-01	1.13e-05*	2.44e-12*
Temporal	2.48e-02	1.21e-02	4.02e-11*
Frontal	2.91e-02	6.26e-03	2.12e-01
Parietal	4.22e-01	2.95e-07*	8.33e-12*

Supplementary table 8. Statistical testing for significant differences in volumes of different brain regions between PCA and tAD at -10, 0 and 10 years from t_0. Shown here are p-values from two-tailed t-tests. (*) Statistically significant differences at significance level = 2.08e-3, Bonferroni corrected for all 28 comparisons.

	Significant differences in DEM estimated ROI volumes		Significant differences in EBM estimated abnormality sequence	
Region	years from t_0	Estimated volume PCA<tAD tAD<PCA	Subregion	Estimated earlier abnormality PCA<tAD tAD<PCA
occipital	-10	-	inferior	PCA< ***
	0	PCA< *	middle	PCA< ***
	10	PCA< **	superior	PCA< ***
			pole	tAD < ***
			occipital fusif.	PCA< ***
			cuneus	PCA< ***
			calcarine	-
			lingual	-
parietal	-10	-	superior	PCA< ***
	0	PCA< **	angular	tAD < ***
	10	PCA< ***	precuneus	tAD < ***
			supra-marginal	-
			postcentral	PCA< ***
temporal	-10	-	superior	-
	0	-	middle	tAD < ***
	10	PCA< ***	inferior	tAD < ***
			pole	-
frontal	-10	-	superior	-
	0	-	middle	tAD < ***
	10	-	inferior	tAD < ***
			precentral	-
hippocampus	-10	tAD < ***	hippocampus	tAD < ***
	0	tAD < ***		
	10	tAD < *		

entorhinal	-10	tAD < **	entorhinal	tAD < ***
	0	tAD < ***		
	10	-		
ventricles	-10	tAD < *	ventricles	PCA < ***
	0	tAD < *		
	10	PCA < ***		

Supplementary Table 9. Summary of the testing for statistically significant differences in neuroimaging between posterior cortical atrophy vs typical Alzheimer's disease. For each brain region, we show on the left differences between estimated biomarker trajectories of posterior cortical atrophy vs typical Alzheimer's disease at -10, 0 and 10 years from t_0. On the right half of the table, we show for key subregions statistically significant differences of how early each biomarker becomes abnormal in the EBM-estimated sequences. We denote by PCA < : posterior cortical atrophy (PCA) < typical Alzheimer's disease (tAD) either that the ROI volume is lower in the posterior cortical atrophy cohort (for DEM, left side) or that volume loss is earlier in the posterior cortical atrophy cohort (for EBM, right side) for that subregion, relative to the other subregions. We denote statistical significance as follows: (-) no statistically significant differences under Bonferroni corrections (DEM: p > 2e-03, EBM: p > 2e-04), (*) p < 2e-03/2e-04, (**) p < 1e-06, (***) p < 1e-09.

Neuropsychology supplementary results

Ordering of abnormality – Event Based Model

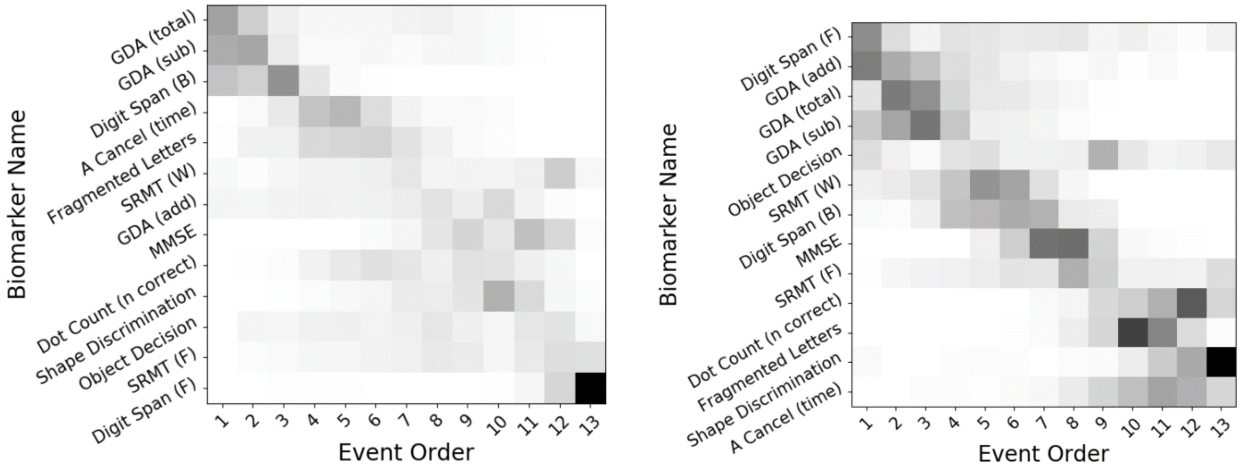

Supplementary Figure 5: Bootstrap samples of the cognitive sequence as estimated by the event-based model, for the PCA (left) and typical AD (right) cohorts.

Continuous Trajectory Estimation – Differential Equation Model

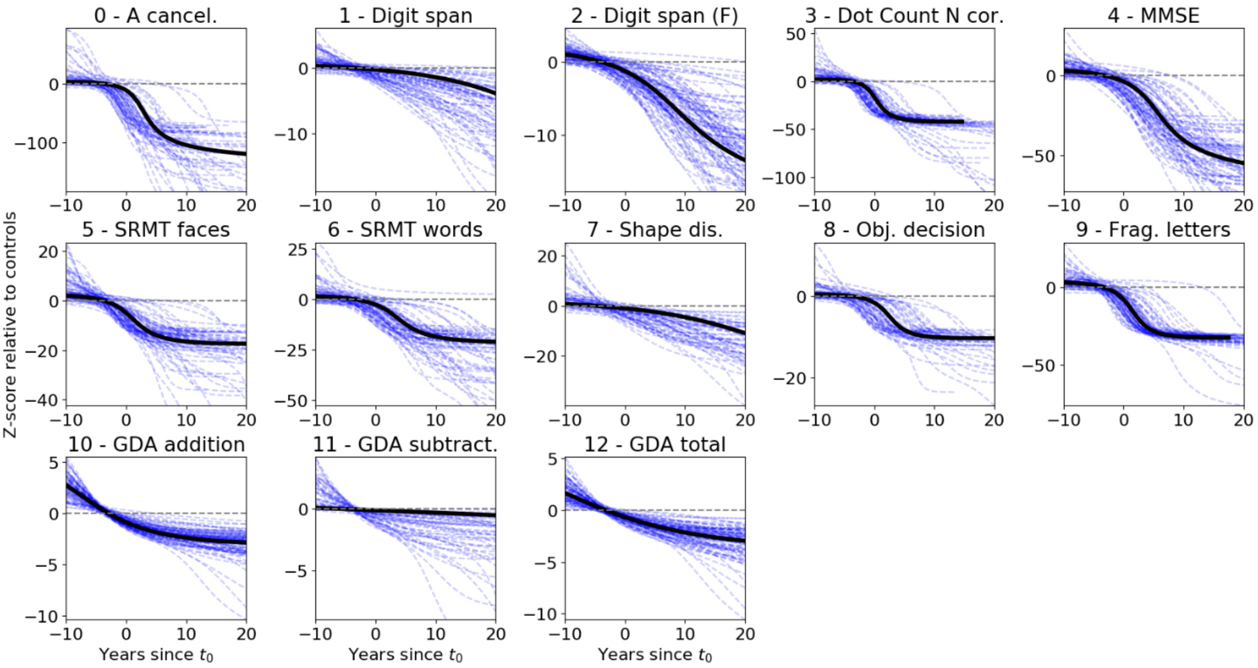

Supplementary Figure 6. Mean trajectories for cognitive tests for PCA with samples from the posterior distribution showing the confidence of the mean trajectory. The axis shows the number of years since baseline visit, and the y-axis shows the test score.

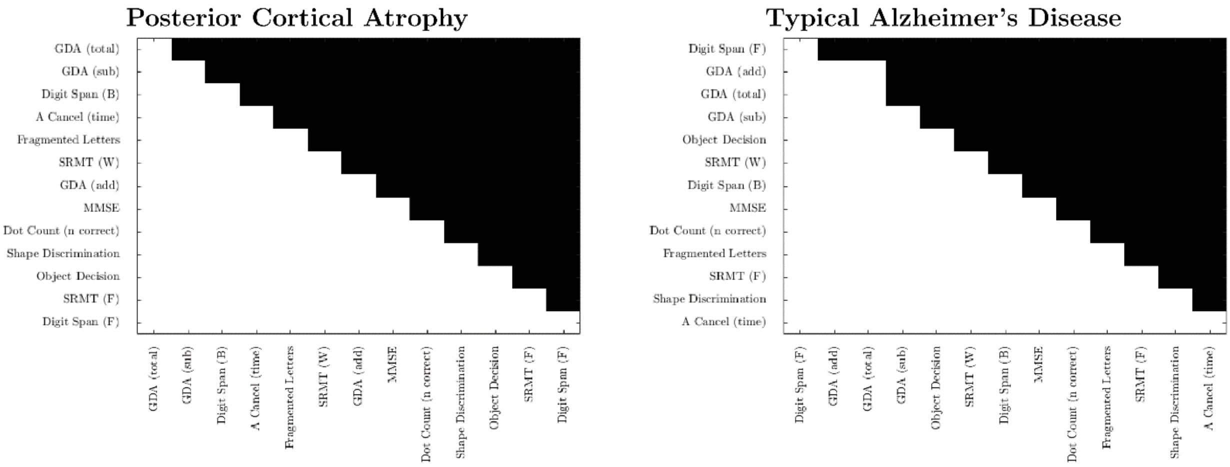

Supplementary Figure 7: Hypothesis testing of significant differences in the EBM-ordering of cognitive events for PCA (left) and tAD (right). See Supplementary Figure 3 for details on hypothesis tests used. Black squares represent a pair of biomarkers *(X, Y)* for which the position of biomarker X within the sequence is significantly smaller (null hypothesis rejected) than the position of biomarker Y. Given most squares above the diagonal are black, we are confident that the biomarkers in PCA and tAD become abnormal in this ordering.

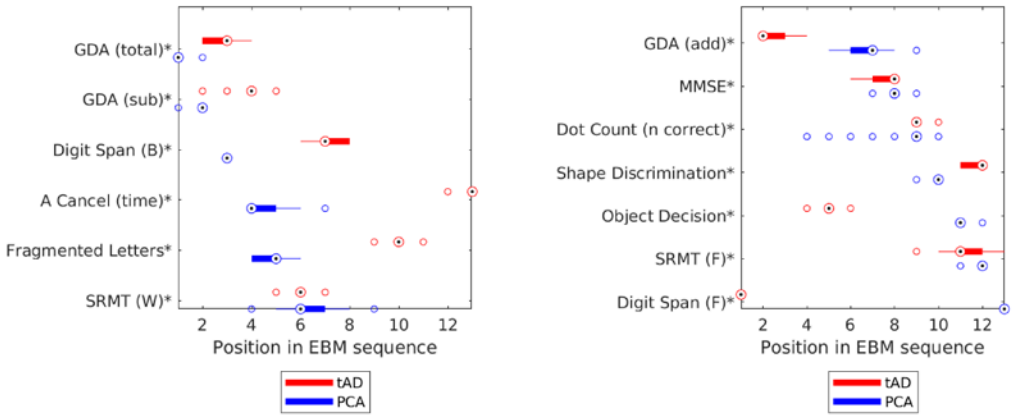

Supplementary Figure 8. Statistical testing of significant differences in positions of each biomarker for PCA vs tAD. See Supplementary Figure 7 for full details on statistical testing. (*) Statistically significant differences according to non-parametric Mann-Whitney U test, Bonferroni corrected ($p < 7.14e-04$). All biomarkers show significant differences in positions within PCA and tAD abnormality sequences.

Experiments with participants with molecular or pathological evidence for underlying AD

We also re-analysed the subset of patients which had their diagnosis supported by pathological or molecular evidence of AD. The results, shown in the figures below, are similar to the results using the full set of subjects, supporting the validity of the results based on the overall sample.

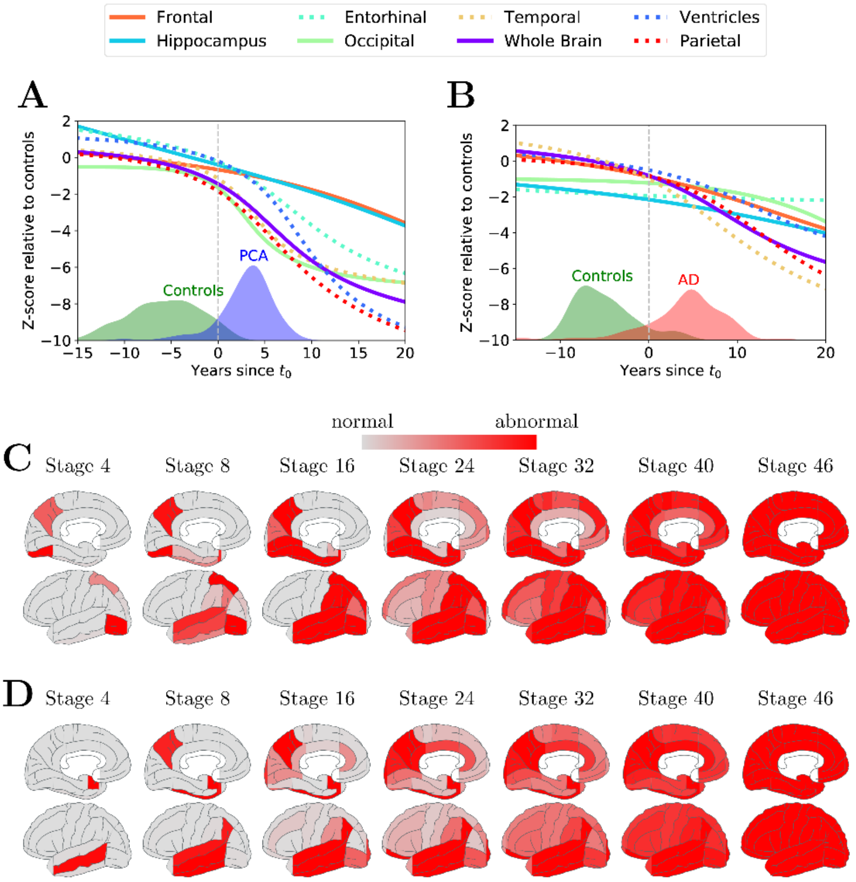

Supplementary Figure 9: (A-B) Longitudinal DEM analysis of neuroimaging data in PCA and tAD patients in whom molecular of pathological evidence for underlying AD pathology was available. (C-D) Cross-sectional EBM analysis of neuroimaging data for (C) PCA and (D) tAD groups in whom molecular of pathological evidence for underlying AD pathology was available. Results are similar to those obtained in Figure 2 from the main manuscript.

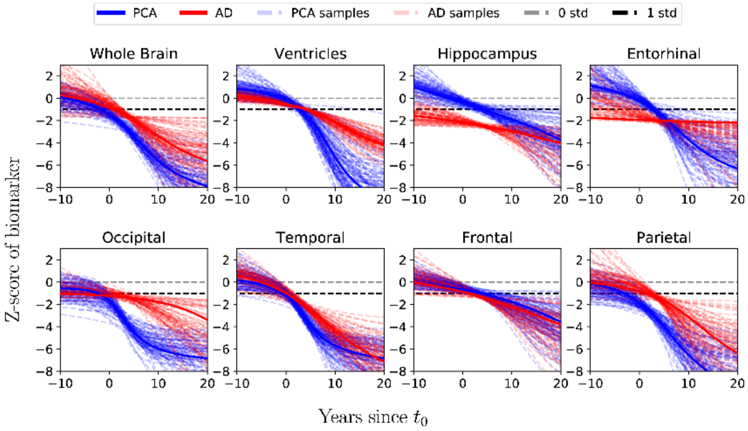

Supplementary Figure 10: Neuroimaging analysis for the subset of patients in whom molecular of pathological evidence for underlying AD pathology was available. Results are similar to the ones of Figure 5 in the main manuscript.

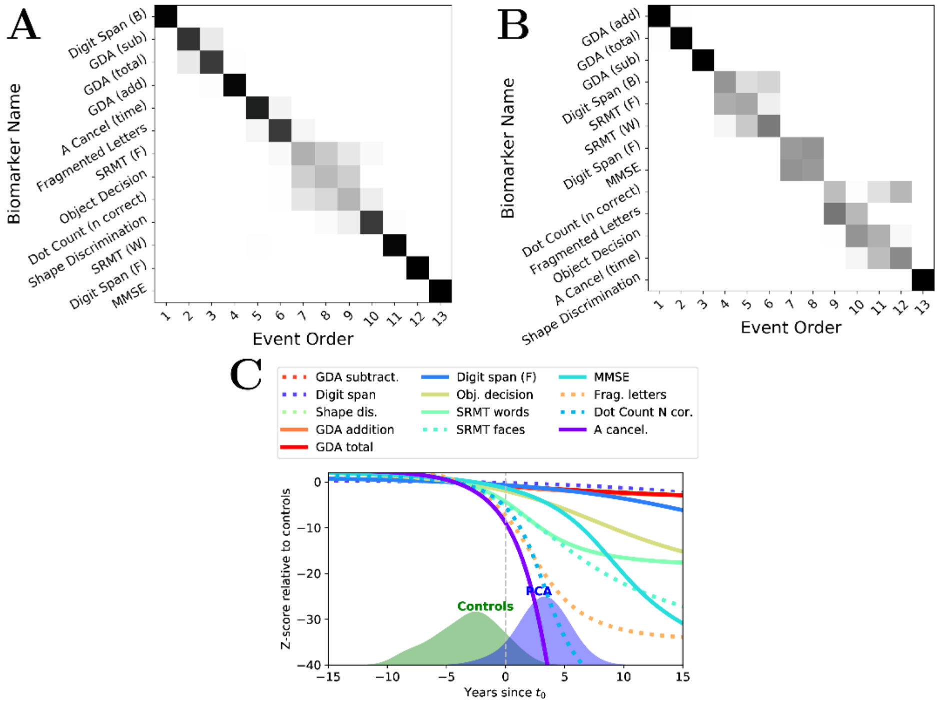

Supplementary Figure 11: (A-B) Cross-sectional EBM analysis of neuropsychological data for (A) PCA and (B) tAD groups in whom molecular of pathological evidence for underlying AD pathology was available. (C) Longitudinal DEM analysis of neuroimaging data in PCA patients in whom molecular of pathological evidence for underlying AD pathology was available. Results are similar to those obtained in Figure 6 from the main manuscript.

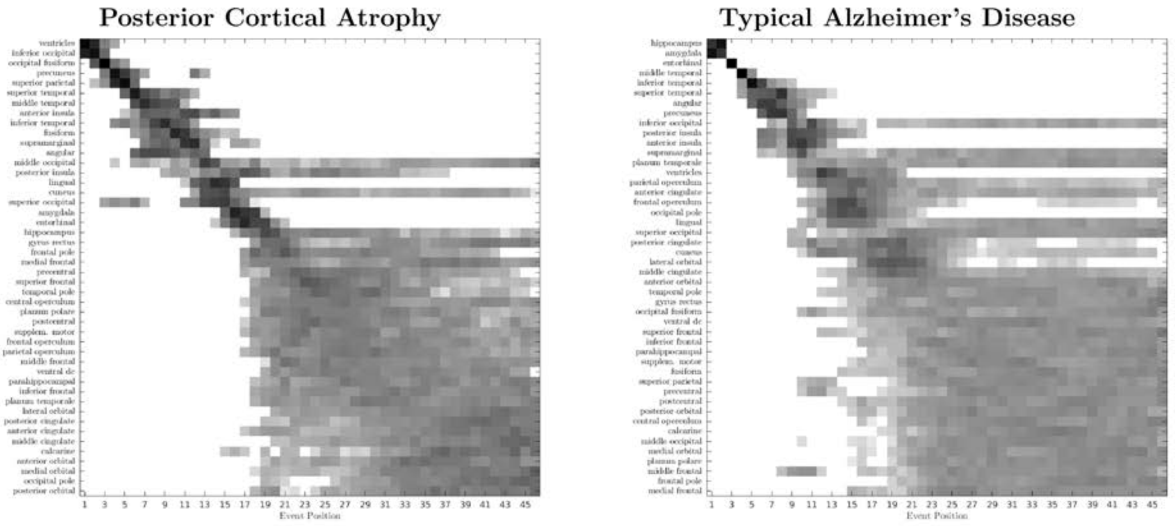

Supplementary Figure 12: For subjects with diagnosis supported by molecular and pathological evidence, we show the positional variance diagram of the most likely atrophy sequence, as estimated by the event-based model, for (left) PCA progression and (right) tAD progression.

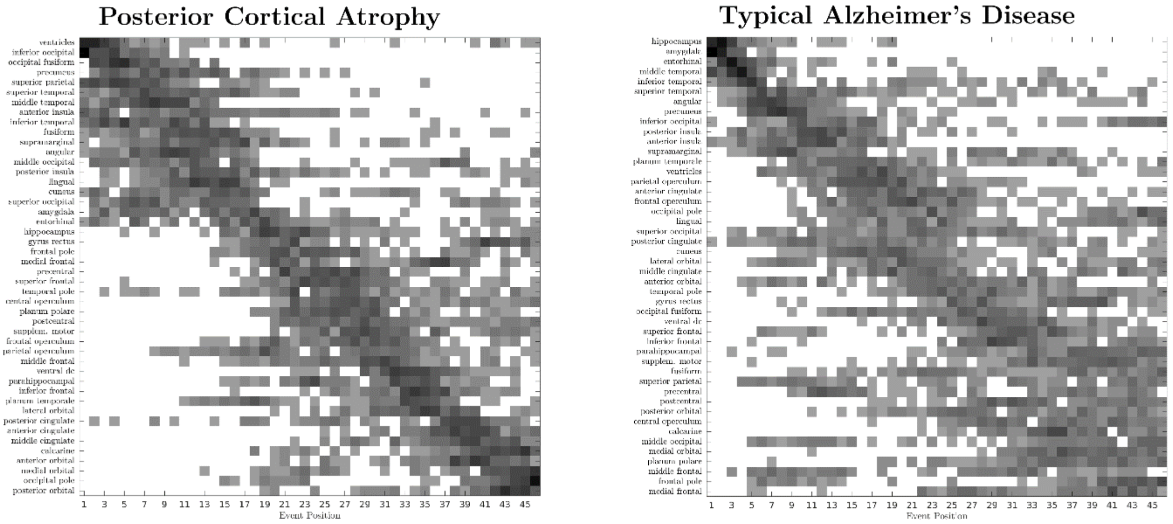

Supplementary Figure 13: For subjects with diagnosis supported by molecular and pathological evidence, we show bootstrap samples of the atrophy sequence as estimated by the event-based model, for the PCA and typical AD cohorts.

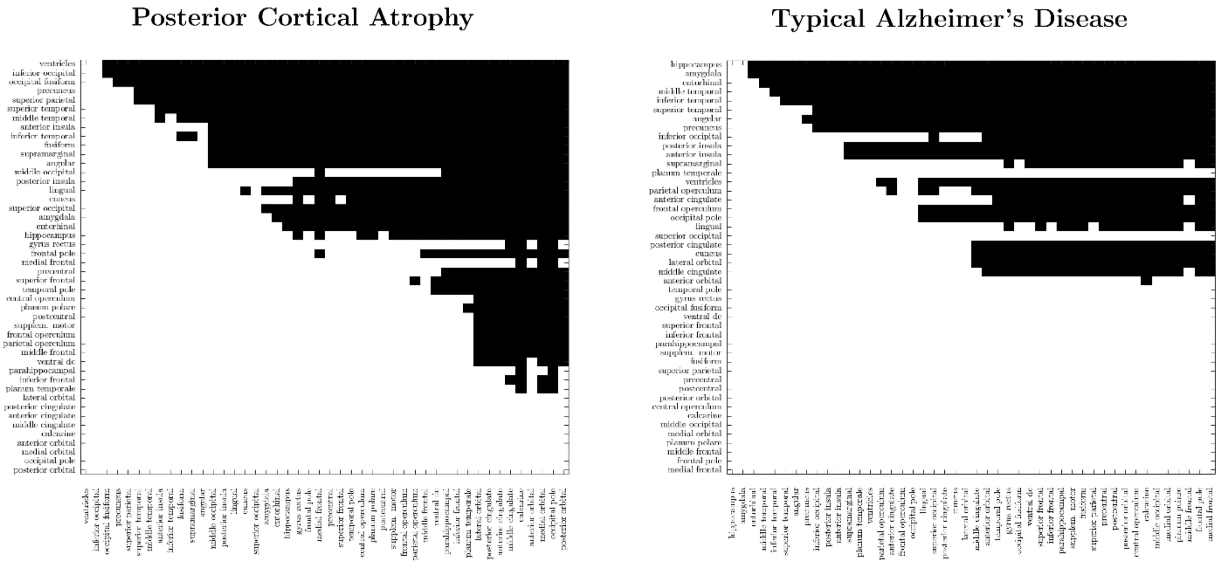

Supplementary Figure 14: For subjects with diagnosis supported by molecular and pathological evidence, we show the hypothesis testing for significant differences in the ordering of events within PCA (left) and typical AD (right). See Supplementary Figure 3 for details on the statistical test used.

Region	T1 = -10 years	T2 = 0 years	T3 = 10 years
Whole Brain	9.59e-03	1.38e-27*	2.76e-28*
Ventricles	2.02e-11*	2.93e-14*	3.26e-34*
Hippocampus	8.43e-35*	1.02e-75*	1.12e-36*
Entorhinal	8.32e-05*	1.03e-33*	4.64e-03
Occipital	5.16e-01	2.09e-18*	2.67e-39*
Temporal	1.49e-02	3.85e-01	1.75e-31*
Frontal	6.79e-01	3.91e-06*	3.04e-05*
Parietal	1.17e-03*	1.79e-40*	1.73e-22*

Supplementary table 15: For subjects who had their diagnosis supported by molecular and pathological biomarkers, we show statistical testing for significant differences in volumes of different brain regions between PCA and tAD at -10, 0 and 10 years from t_0. Shown here are p-values from two-tailed t-tests. (*) Statistically significant differences at significance level = 2.08e-3, Bonferroni corrected for all 28 comparisons.

Region	Whole Brain	Ventricles	Hippocampus	Entorhinal	Occipital	Temporal	Frontal	Parietal
Whole Brain	-	-	-	-	-	-	-	-
Ventricles	7.65e-12*	-	-	-	-	-	-	-
Hippocampus	1.46e-12*	8.26e-01	-	-	-	-	-	-
Entorhinal	1.10e-12*	2.04e-02	1.18e-02	-	-	-	-	-
Occipital	5.56e-10*	2.62e-24*	1.02e-25*	2.93e-21*	-	-	-	-
Temporal	2.89e-01	4.36e-08*	2.13e-08*	1.28e-09*	2.41e-10*	-	-	-
Frontal	6.06e-01	1.42e-11*	1.76e-12*	1.19e-11*	2.85e-08*	1.44e-01	-	-
Parietal	1.58e-03*	1.32e-15*	4.47e-16*	9.32e-16*	2.60e-02	2.82e-04*	9.13e-03	-

Supplementary table 16: For subjects with diagnosis supported by molecular and pathological evidence, we show statistical testing for significant differences in volumes of brain regions for PCA subjects at -10 years before disease t_0. Shown here are p-values from two-tailed t-tests. (*) Statistically significant differences at significance level = 1.78e-3, Bonferroni corrected for all 28 comparisons.

Region	Whole Brain	Ventricles	Hippocampus	Entorhinal	Occipital	Temporal	Frontal	Parietal
Whole Brain	-	-	-	-	-	-	-	-
Ventricles	1.98e-70*	-	-	-	-	-	-	-
Hippocampus	4.10e-60*	4.28e-04*	-	-	-	-	-	-
Entorhinal	1.92e-62*	9.14e-01	1.11e-03*	-	-	-	-	-
Occipital	2.13e-08*	1.80e-62*	1.00e-54*	7.72e-57*	-	-	-	-
Temporal	1.07e-08*	1.81e-46*	2.14e-36*	9.81e-41*	4.78e-18*	-	-	-
Frontal	3.36e-45*	4.70e-19*	4.39e-09*	7.96e-16*	1.75e-41*	7.30e-22*	-	-
Parietal	1.70e-14*	1.23e-73*	1.40e-65*	5.58e-67*	1.36e-01	9.85e-26*	6.96e-52*	-

Supplementary table 17: For subjects with diagnosis supported by molecular and pathological evidence, we show statistical testing for significant differences in volumes of brain regions for PCA subjects at disease t_0. See Supplementary Figure 14 for more details on statistical testing.

Region	Whole Brain	Ventricles	Hippocampus	Entorhinal	Occipital	Temporal	Frontal	Parietal
Whole Brain	-	-	-	-	-	-	-	-
Ventricles	8.12e-07*	-	-	-	-	-	-	-
Hippocampus	8.22e-83*	1.32e-37*	-	-	-	-	-	-
Entorhinal	9.76e-23*	4.22e-06*	1.96e-20*	-	-	-	-	-
Occipital	5.69e-05*	4.11e-12*	1.83e-63*	3.57e-25*	-	-	-	-
Temporal	3.74e-03	6.51e-11*	3.62e-88*	1.69e-27*	5.01e-02	-	-	-
Frontal	3.24e-72*	3.08e-32*	8.06e-01	3.34e-17*	2.52e-55*	6.99e-77*	-	-
Parietal	3.25e-06*	1.18e-13*	1.37e-64*	6.77e-27*	5.53e-01	8.73e-03	2.20e-56*	-

Supplementary table 18: For subjects with diagnosis supported by molecular and pathological evidence, we show statistical testing for significant differences in volumes of brain regions for PCA subjects at 10 years after disease t_0. See Supplementary Figure 14 for more details on statistical testing.

Region	Whole Brain	Ventricles	Hippocampus	Entorhinal	Occipital	Temporal	Frontal	Parietal
Whole Brain	-	-	-	-	-	-	-	-
Ventricles	5.29e-02	-	-	-	-	-	-	-
Hippocampus	3.27e-19*	6.18e-38*	-	-	-	-	-	-
Entorhinal	2.68e-01	8.10e-01	1.49e-08*	-	-	-	-	-
Occipital	1.30e-10*	1.20e-22*	3.54e-06*	3.10e-04*	-	-	-	-
Temporal	7.87e-01	1.16e-03*	2.54e-30*	1.17e-01	2.13e-18*	-	-	-
Frontal	3.38e-03	1.26e-02	7.05e-19*	4.31e-01	4.20e-08*	2.66e-05*	-	-
Parietal	6.08e-01	4.03e-01	2.92e-12*	5.69e-01	2.72e-06*	4.17e-01	7.29e-02	-

Supplementary table 19: For subjects with diagnosis supported by molecular and pathological evidence, we show statistical testing for significant differences in volumes of brain regions for tAD subjects at 10 years before disease t_0. See Supplementary Figure 14 for more details on statistical testing.

Region	Whole Brain	Ventricles	Hippocampus	Entorhinal	Occipital	Temporal	Frontal	Parietal
Whole Brain	-	-	-	-	-	-	-	-
Ventricles	8.66e-07*	-	-	-	-	-	-	-
Hippocampus	1.39e-46*	2.06e-94*	-	-	-	-	-	-
Entorhinal	1.69e-13*	2.33e-40*	6.05e-14*	-	-	-	-	-
Occipital	1.76e-05*	1.00e-28*	4.43e-36*	1.79e-06*	-	-	-	-
Temporal	1.21e-09*	7.29e-37*	5.71e-40*	1.17e-05*	2.08e-01	-	-	-
Frontal	8.74e-02	1.11e-18*	1.07e-52*	2.84e-14*	4.58e-04*	2.32e-07*	-	-
Parietal	4.41e-01	2.10e-04*	8.36e-45*	8.75e-14*	1.84e-06*	6.24e-11*	1.35e-02	-

Supplementary table 20: For subjects with diagnosis supported by molecular and pathological evidence, we show statistical testing for significant differences in volumes of brain regions for tAD subjects at disease t_0. See Supplementary Figure 14 for more details on statistical testing.

Region	Whole Brain	Ventricles	Hippocampus	Entorhinal	Occipital	Temporal	Frontal	Parietal
Whole Brain	-	-	-	-	-	-	-	-
Ventricles	1.79e-31*	-	-	-	-	-	-	-
Hippocampus	1.31e-02	7.57e-39*	-	-	-	-	-	-
Entorhinal	1.76e-06*	1.26e-14*	4.00e-06*	-	-	-	-	-
Occipital	1.25e-14*	1.27e-02	1.06e-12*	3.34e-03	-	-	-	-
Temporal	1.48e-04*	2.17e-60*	1.45e-12*	2.59e-18*	1.18e-30*	-	-	-
Frontal	2.93e-22*	1.24e-01	3.09e-22*	1.61e-06*	2.90e-01	3.13e-44*	-	-
Parietal	6.14e-01	8.64e-21*	2.44e-02	1.48e-04*	2.87e-10*	1.81e-02	3.45e-15*	-

Supplementary table 21: For subjects with diagnosis supported by molecular and pathological evidence, we show statistical testing for significant differences in volumes of brain regions for tAD subjects at 10 years after disease t_0. See Supplementary Figure 14 for more details on statistical testing.

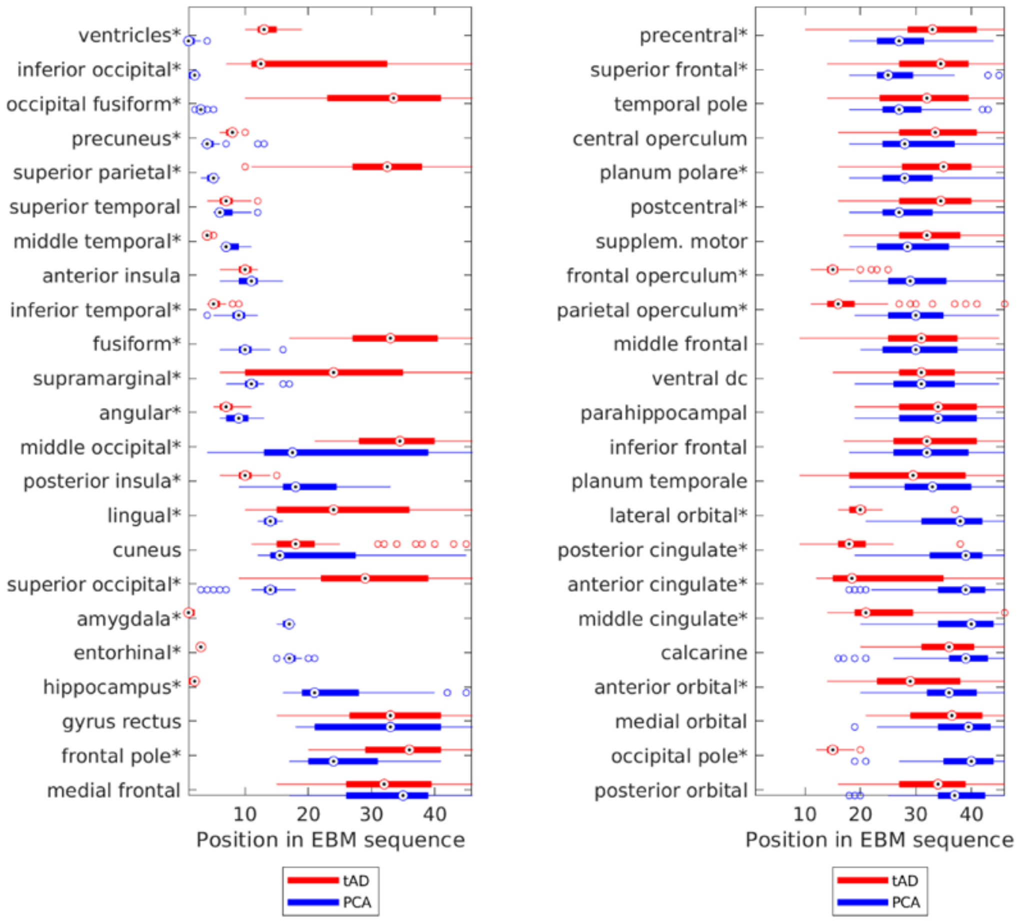

Supplementary Figure 15: For subjects who had their diagnosis supported by molecular or pathological biomarkers, we show statistically significant differences in positions of each biomarker in the EBM abnormality sequences, for both PCA and typical AD. (*) Statistically significant differences in position of a biomarker in the EBM sequences for PCA and tAD at 95% confidence, Bonferroni corrected for multiple comparisons (significance level = 2e-04). A non-parametric Mann-Whitney U test has been applied because of non-gaussianity of the data, which represents discrete ranks in a sequence. All biomarkers apart from lingual show significant differences – it is likely that there are differences in atrophy progression between PCA and tAD.

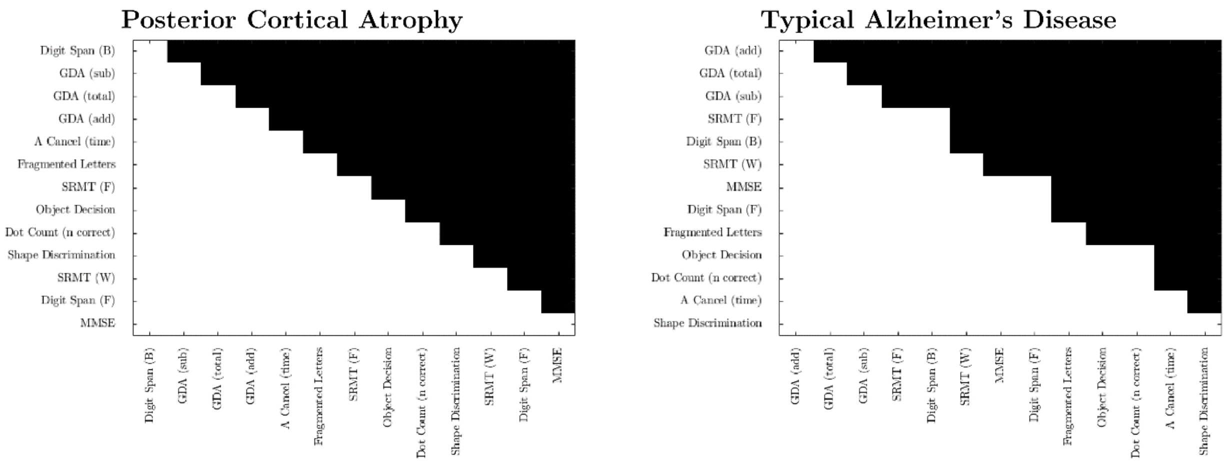

Supplementary Figure 16: For subjects which had their diagnosis confirmed by molecular/CSF biomarkers, we show hypothesis testing of significant differences in the EBM-ordering of cognitive

events for PCA (left) and tAD (right). See Supplementary Figure 3 for details on hypothesis tests used. Black squares represent a pair of biomarkers *(X, Y)* for which the position of biomarker X within the sequence is significantly smaller (null hypothesis rejected) than the position of biomarker Y. Given most squares above the diagonal are black, we are confident that the biomarkers in PCA and tAD become abnormal in this particular order.

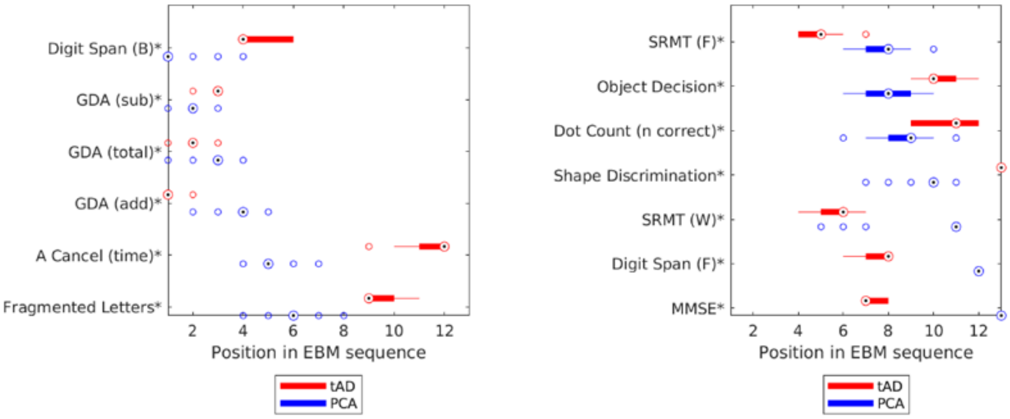

Supplementary Figure 17: For subjects which had their diagnosis confirmed by molecular/CSF biomarkers, we show the statistical testing of significant differences in positions of each biomarker within the EBM-estimated sequences, for PCA vs tAD. See Supplementary Figure 7 for full details on statistical testing. (*) Statistically significant differences according to non-parametric Mann-Whitney U test, Bonferroni corrected ($p < 7.14e-04$). All biomarkers show significant differences in positions within PCA and tAD abnormality sequences.

References

[1] H. M. J. Fonteijn, M. . Modat, M. J. Clarkson, J. . Barnes, M. . Lehmann, N. Z. Hobbs, R. I. Scahill, S. J. Tabrizi, S. . Ourselin, N. C. Fox and D. C. Alexander, “An event-based model for disease progression and its application in familial Alzheimer's disease and Huntington's disease,” *NeuroImage,* vol. 60, no. 3, pp. 1880-1889, 2012.

[2] J. W. Ashford and F. A. Schmitt, “Modeling the time-course of Alzheimer dementia,” *Current Psychiatry Reports,* vol. 3, no. 1, pp. 20-28, 2001.

[3] V. L. Villemagne, S. . Burnham, P. . Bourgeat, B. . Brown, K. A. Ellis, O. . Salvado, C. . Szoeke, S. L. Macaulay, R. N. Martins, P. . Maruff, D. . Ames, C. C. Rowe and C. L. Masters, “Amyloid β deposition, neurodegeneration, and cognitive decline in sporadic Alzheimer's disease: a prospective cohort study,” *Lancet Neurology,* vol. 12, no. 4, pp. 357-367, 2013.

[4] N. P. Oxtoby, A. L. Young, D. M. Cash, T. L. S. Benzinger, A. M. Fagan, J. C. Morris, R. J. Bateman, N. C. Fox, J. M. Schott and D. C. Alexander, “Data-driven models of dominantly-inherited Alzheimer’s disease progression,” *Brain,* 2018.
